# Withstand control: standing posture differentially affects space-based and feature-based cognitive control through enhanced physiological arousal

**DOI:** 10.1038/s41598-025-11692-6

**Published:** 2025-07-20

**Authors:** Nuno Busch, Artyom Zinchenko, Martin Halle, Thomas Geyer

**Affiliations:** 1 School of Management, Technische Universität München, 80333 Munich, Germany; 2 Department of Psychology, Ludwig-Maximilians-Universität München, 80802 Munich, Germany; 3 Munich Center for NeuroSciences – Brain & Mind, 82152 Planegg-Martinsried, Germany; 4 NICUM – NeuroImaging Core Unit Munich, 80336 Munich, Germany; 5 Department of Preventive Sports Medicine and Sports Cardiology, Technische Universität München, 80333 Munich, Germany; 6 DZHK (German Center for Cardiovascular Research), Partner Site Munich Heart, Munich, 80636 Germany

**Keywords:** Arousal, Attentional control, Body posture, Cognitive control, Executive function, HRV, Inhibition, Sit-stand desk, Human behaviour, Psychology, Cardiology, Cardiovascular biology, Neurophysiology

## Abstract

Can simply standing upright sharpen our minds? Whereas initial works proposed that standing posture reduces distractor interference in the Stroop task, subsequent replication attempts yielded mixed results. Importantly, previous studies mostly ignored individual fitness factors like BMI, physical activity level, and cardiac measures, which have recently been suggested to explain mixed findings. Here, we addressed these inconsistencies by controlling for such fitness characteristics in *N* = 36 healthy adults. Additionally, we expanded upon previous research by examining posture’s influence on previously unexplored space-based cognitive control using the Navon task. We found evidence *against* an effect of standing posture on Stroop interference, while, in contrast, standing posture significantly improved spatial conflict processing in the Navon task. Most importantly, this effect was fully mediated by enhanced physiological arousal as indexed by reduced HRV, directly supporting the proposed mechanism of how standing influences cognitive control through increased postural demands. Our findings challenge the idea of a unified attentional control system, revealing that posture differentially influences conflict processing depending on its task-specific nature. These insights may advance theoretical understanding and have practical implications: in increasingly sedentary societies, simple changes like standing could selectively enhance certain cognitive functions, improving effective work and learning environments.

**Supplementary Information:**

The online version contains supplementary material available at 10.1038/s41598-025-11692-6.

## Introduction

Over the last few years, evidence has shown that physical activity and body posture, such as standing, sitting, or lying down, influence cognitive functioning. This growing awareness has increased commercial interest in height-adjustable work desks, advertised to improve not only physical health but also work productivity while standing^1–3^. Interestingly, previous research has indeed suggested that standing posture can affect various cognitive processes, including attention and visual search^4–7^ alertness^8^ and memory^9–11^.

Even though posture does seem to modulate attention, multiple attention functions and associated paradigms exist ^[e.g.,12^, which raises the question of whether posture influences specific (e.g., *selective)* attention functions, such as *inhibitory cognitive control.* Inhibitory cognitive control (sometimes also termed inhibition^13^) is one of humans’ most prominent executive functions, alongside mental set shifting and information updating^13,14^. It describes the ability of target monitoring and associated mental resource recruitment to focus on target-relevant aspects selectively and to inhibit irrelevant or distracting information, essential for successful performance on a wide range of everyday cognitive tasks, be it during reading, problem-solving, or decision-making. Initial work has proposed that cognitive control can also be influenced by bodily factors, including standing posture^6,15^.

One prominent hypothesis suggests that attentional selectivity is improved through the additional attentional demands of postural maintenance during standing^15, see also 16^. Assuming shared limited resources between cognitive and motor functions^10,17,18^ the increased demand for postural control should allow less processing of distracting information^6,15,19^. This hypothesis aligns with the Load Theory of attention, which proposes that resource-demanding manipulations (such as those with high perceptual load) diminish distractor interference^20^. In support of this view, neuroimaging studies have shown increased frontal lobe activation during standing^21,22^ which overlaps with fronto-parietal attention network structures^23^ particularly involved in inhibitory cognitive control^24^. Recent empirical evidence lends support to this theoretical framework: Johannsen et al. (2023) demonstrated specific time-locked balance correlates of cognitive conflict during standing (reduced postural sway variability in incongruent conflict trials^25^), suggesting that cognitive demands may be associated with differential postural behavior. However, the relationship between postural demand and cognitive performance may not be linear. Smith et al. proposed that simple standing might increase arousal from low to moderate levels and thus optimize cognitive resources and enhance selective attention^6^ though more demanding postural situations might decrease cognitive performance due to the excessive consumption of shared attentional resources^10^. Together, cognitive control may be enhanced during standing through the mechanism of heightened physiological arousal accompanying the additional attentional demands for postural control^[cf.26^.

The first two studies investigating postural modulation of cognitive control indeed found improved control ability (reduced Stroop interference effect) during standing as opposed to sitting position^6,15^ supported by a third work demonstrating a similar pattern (higher Stroop accuracy during standing) in Japanese students^27^. In contrast, the positive association between standing and cognitive control has been questioned by other researchers who could not demonstrate the same effects in replication studies^5,19,28^ of Rosenbaum et al.’s initial experiments^15^. Similarly, Caron and colleagues^29^ found no modulation of Stroop control by bodily posture, regardless of additional balance task loads (standing on one vs. two feet) or response mode (vocal vs. tactile).

In summary, the evidence supporting the initial findings and theorized mechanisms remains inconclusive. Recent meta-analytic reviews synthesizing the limited number of reported effect sizes cast doubt on the significance of body posture’s influence on cognitive control, revealing only a non-significant trend towards enhanced selective attention during standing posture^30^. Consequently, while the relationship between posture and cognitive control is intriguing, its robustness and generalizability require further investigation.

*Possible reasons for inconclusive findings*. Previously, it has been suggested that the inconsistency in findings on the *Stroop x posture* interaction may have been due to subtle factors, such as sample characteristics of participant populations, that have not yet been accounted for^19,29^. This is important since the load induced by standing posture may differ highly between individuals. Depending on - for instance - their age, physical features, or fitness status, individuals may or may not stand with ease for a longer period or potentially struggle to keep the balance if they are old, obese, or unfit^19,31,32^. In consequence, depending on the individual characteristics that influence the postural load experienced by a person, standing posture may have a positive, detrimental, or no effect on cognitive control.

To our knowledge, no study has yet accounted for individual differences of participants that may influence the existence (or absence) of the posture effect. Therefore, the first aim of the current study was to clarify the relationship between bodily posture and Stroop control, considering a variety of personal physical measures that may influence the effects of body posture and may thus have contributed to the conflicting results reported previously. Instead of adapting postural load conditions individually on the participant’s age and fitness status (as previously proposed^19^), here we took the alternative approach, using individual physical measures such as body mass index (BMI), self-reported individual physical activity, and state-dependent physiological measures (cardiac responses) to statistically account for condition-specific postural demands in our analysis.

*The Stroop task as a measure of cognitive control.* It is also important to note that most previous studies have focused solely on one measure of cognitive control: the Stroop effect. In the Stroop task^33^ participants name the ink color of printed color words while inhibiting the interfering color name. Whereas the Stroop task is undoubtedly a classic measure of cognitive control, it is not the sole or necessarily the best measure of (inhibitory) cognitive control ^[e.g.,13^. Other tasks assessing cognitive control or selective attention include the flanker, Navon, and Simon tasks^14,30,34–37^. Importantly, each of these tasks also measures substantial task-specific abilities. For example, it has been argued that Stroop and Navon tasks differ substantially in their task-specific demands and their underlying neurocognitive mechanisms, although both tasks can be applied to measure inhibitory control of interfering information^38,39^. Notably, specifically the Stroop task often behaves differently than other measures of inhibition^[e.g.,40^ and doesn’t always correlate with performance in other inhibition tasks^14,38^. This underscores the importance of considering multiple measures when assessing cognitive control and how it is affected by body posture.

To our knowledge, only preliminary evidence exists from few very recent studies on posture effects that applied inhibitory cognitive control measures other than the Stroop task. Three of them adopted variants of the flanker task^4,41,42^ where participants must inhibit task-irrelevant arrows surrounding a target arrow while indicating the target arrow’s direction. These studies did not find significant differences in interference control between standing and sitting. A fourth study applied the Simon task, interpreting it as a measure of cognitive conflict at the response selection level^25^. This study found a significant interaction of posture and congruency, with an *increased*(!) reaction time (RT) congruency effect (i.e., worse conflict processing) during standing.

Together, the previous research on the influence of posture on cognitive control has yielded very inconclusive results. Studies have primarily focused on feature-based control in Stroop tasks, with limited exploration of other paradigms like flanker and Simon tasks. Preliminary findings across these inhibitory control tasks show no consistent pattern: Some studies suggest a positive effect of standing on Stroop control^15,30^ while others indicate a negative influence of standing on Simon task performance^25^. In contrast, no significant differences were observed in flanker paradigms^4,41,42^. This variability suggests that postural demands may differentially affect task-specific cognitive control mechanisms. Consequently, the broader impact of posture on other aspects of cognitive control mechanisms remains elusive and warrants further investigation.

Therefore, the second important aim of the present study was to extend the previous findings by exploring how body posture affects cognitive control in another prominent cognitive control task - the Navon Task^36^. The Navon task measures the ability to selectively attend to a global or local stimulus level: Participants are presented with large letters made up of smaller letters, inducing perceptual conflicts between automized processing of global stimuli and local stimulus processing. Thus, the Navon and Stroop tasks assess two qualitatively different aspects of cognitive control, i.e., feature-based (Stroop) and space-based (Navon) control, that both require inhibition of task-irrelevant stimulus aspects^38,43,44^. In sum, the present study aimed to directly contrast posture effects in two different facets of cognitive control while testing if such effects are influenced by individual characteristics of physical fitness. Our design allows us to test moderations of the posture effect and even the proposed mediation mechanism through enhanced physiological arousal in case a main effect of posture may be evident.

## Methods

### Participants

41 participants were invited to participate in the study, of which 36 individuals (22 female, 14 male, 4 left-handed) completed both tasks during both (standing vs. sitting) sessions (the remaining individuals dropped out of the study or had incomplete data). Whereas a sample size of *N* = 6–8 was reported to be sufficient to detect an initially proposed effect size of the posture effect^6,45^ with reasonable power^19^, we ensured our sample size was comparable to that in recent studies which failed to replicate the *Stroop x posture* interaction (e.g., *n* = 44 in Exp. 1 and *n* = 38 in Exp. 2 of^19^). All participants were recruited in Munich, Germany, and received monetary compensation or LMU course credit for their participation. Data collection took place between November 2021 and May 2022. Exclusion criteria involved being in poor mental health, being on medication, having physical limitations to sit or stand, or having neurological diseases. All participants had normal or corrected-to-normal vision. The DFG grant project (GE 1889/4 − 2) that this work is associated with was approved by the LMU’s Department of Psychology ethics committee on April 23rd, 2018. Participants signed informed consent before the experiment. All procedures were in accordance with the ethics guidelines from the Declaration of Helsinki.

To retain consistency with most previous studies investigating the *Stroop x posture* interaction (cf. Supplementary Material 3 in^19^), we did not exclude any participants due to the high error rate. However, excluding participants with poor task performance (e.g., > 20% error rate^38^) did not change the pattern of results severely, as demonstrated in an alternative control analysis (see Supplementary Material 2). On average, participants were 24.75 years old (*Min* = 20, *SD* = 4.38, *Max* = 42) and had varying educational backgrounds (Table 1).

**Table 1:** Summary of sociodemographic sample characteristics.

Characteristic		*n*
Gender	Male Female	14 22
Educational level	Vocational baccalaureate diploma High school degree Bachelor’s degree Master’s degree PhD title	1 10 15 9 1
Handedness	Right-handed Left-handed	32 4
Visual aid	Dependent on visual aid No visual aid needed	19 17

The sample consisted of 36 participants.

### Procedure

The present work was part of a larger project: During the experiment, participants performed two cognitive control tasks (the Navon and Stroop task, as described below) and a visual search task (not reported here^[cf.7^). All these tasks were performed twice in a counterbalanced order: One session was performed in a sitting position, and the other one standing. Within participants, the daytime of the testing was held constant between sessions, with sessions being conducted ideally two days apart (however, the exact interval varied depending on the individual availability of participants, *M*_interval_ = 2.59 days, *SD*_interval_ = 1.22 days). Similarly, the order in which the tasks were performed was counterbalanced between participants but was held constant within participants across the two sessions. Before the experimental tasks in the first testing session, participants completed a questionnaire that included items on demographic variables, questions on personal characteristics (e.g., to calculate BMI), and the short version of the International Physical Activity Questionnaire (IPAQ)^46^. The study was not preregistered.

### Stroop task

**Fig. 1:**
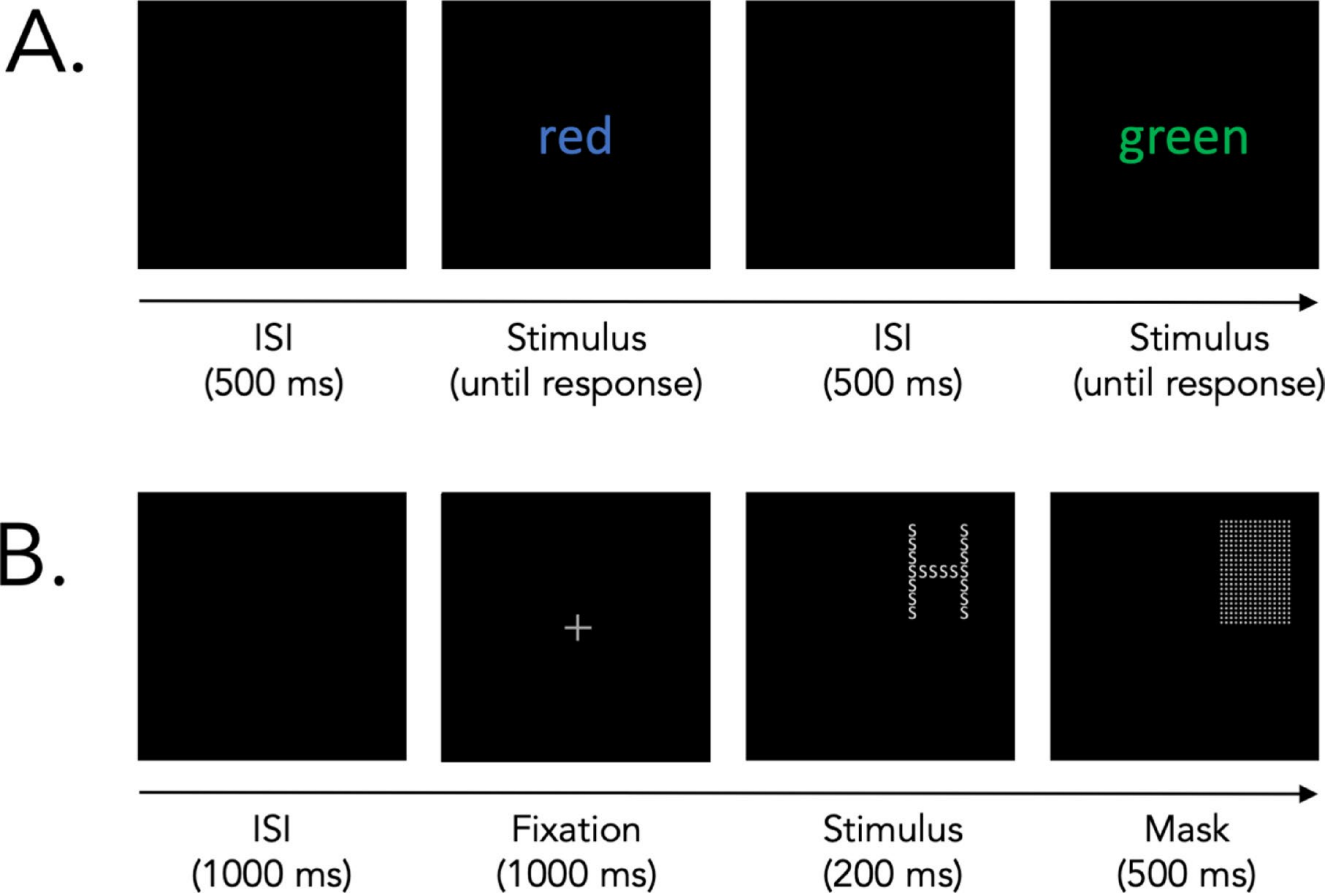
Trial sequences of cognitive control tasks. (**A**) An exemplary trial sequence in an incongruent trial and a congruent trial in the Stroop task. Participants were required to indicate the ink color as quickly and correctly as possible, while inhibiting the word’s meaning. (**B**) Exemplary procedure of an incongruent trial in the Navon task. Notably, the location of stimulus appearance was evenly distributed across trials. Participants were required to indicate the local letters (here: S) as quickly and as correctly as possible, while inhibiting the global letter (here: H). Response time in the Navon task was constrained to 7000 ms after the stimulus onset. ISI = Interstimulus Interval. The figure was copied from Busch et al.^38^ with permission from the authors.

Aiming to replicate previous studies^15,19,29^ we used a classical Stroop task^33^ that challenges individuals to prioritize color recognition over automated reading habits (Fig. 1A). Our task version utilized the three words red, green, and blue. These words were presented in an equally probable distribution of the same target colors - red, green, and blue. The task instruction was to respond as quickly and correctly as possible to the color of the ink by using the arrow keys (left, down, right) with their dominant hand. The arrangement between stimuli and response was kept consistent for all participants. Each word remained visible until a response was submitted without any time constraints. Once a response was recorded, an interstimulus interval (ISI) of 500 ms preceded the presentation of the subsequent word. The task included 84 trials (50% incongruent). To ensure participants were comfortable with the task protocol, they first completed 12 practice trials that were not included in the analyses. During this preliminary phase, feedback was given for both correct and incorrect responses.

### Navon task

In contrast to the Stroop task, the Navon task offers an evaluation of space-based perceptual control abilities. Introduced by Navon^36,^ a consistent observation is that people tend to respond more rapidly to a larger, global stimulus than to a more detailed, local one when dealing with compound, hierarchical stimuli. These stimuli involve a larger letter (global level) made up of smaller letters (local level), which can be either congruent or incongruent (Fig. 1B). In our application of this task, we adhered closely to Navon’s original methodology: To challenge inhibitory cognitive control, participants were required to identify a smaller, local target letter, demanding the suppression of the automatic response to process the larger, global letter. The compound stimuli we employed were the letters “S” and “H”. For instance, an incongruent trial might present a global “S” constructed from numerous smaller “H” letters. Each trial began with an interstimulus interval (ISI) of 1000 ms, followed by the appearance of a centrally positioned fixation cross lasting for 1000 ms. Once the fixation cross vanished, the stimulus letter was displayed in a randomly chosen quadrant of the screen. After 200 ms, the stimulus was masked by a collection of small white dots for 500 ms. Participants had 7000 ms after the stimulus appeared to provide their response. They were prompted to react as rapidly and accurately as possible, using the “S” and “H” keys on the keyboard. The stimulus-response assignment remained consistent for all participants, meaning the “S” and “H” keys always corresponded to their respective letters. This task consisted of 64 trials, evenly balanced between congruent and incongruent, with an equal distribution of letters at both global and local levels across all trials. Before the main task, participants performed 16 practice trials to familiarize themselves with the task dynamics. In this phase, feedback was provided for every response, regardless of its correctness. Practice trials were not included in the analyses.

### Heart rate measurement

During the experiment, heart rate was continuously recorded from a Polar H10 chest strap heart rate sensor (Polar Electro Oy, Kempele, Finland), using the HRV + app (version 2.9.2, ZUZ LLC) on iPhone. After the experiment, the data were downloaded to a computer and processed in R using the RHRV package (R package version 4.2.7)^47^. Following the RHRV package guidelines, we first computed the instantaneous, non-interpolated heart rate (NIHR) before applying the *FilterNIHR()-*function to eliminate outliers and spurious points with unacceptable physiological values from the NIHR time series. Subsequently, the NIHR data was interpolated to create a uniformly sampled HR series for spectral analysis, using the default value of 4 Hz for interpolation frequency. To obtain an individual value for beats per minute (BPM), we extracted the mean NIHR for our manipulation check of how posture influences heart rate. Further, we computed the time domain data to extract the SDNN (standard deviation of RR intervals) as an individual measure of heart rate variability (HRV). Lastly, as an alternative measure of physiological arousal for our control analyses (see Supplementary 3), we performed a frequency analysis to obtain the ratio of power (LF/HF) in the low-frequency band (LF; 0.04–0.15 Hz) vs. the high-frequency band (HF; 0.15–0.4 Hz), using the default values of 300-sec window size and a shift of 30 s for calculating the spectrogram.

These cardiac measures may be particularly useful to index physiological arousal: HRV arises from complex heart-brain interactions and dynamics of the autonomous nervous system – that is, from dynamic interactions between sympathetic and parasympathetic activation, and is (in short recordings) influenced by the parasympathetically-mediated respiratory sinus arrhythmia (RSA). It reflects autonomic balance regulation, vascular tone, and other indices of neurocardiac functioning (for an overview of HRV metrics and their sources, see^48^). While HRV can be strongly affected based on situational context (such as exercise and body posture), a higher vagally-mediated HRV at rest has also been linked to prefrontal cortex functions such as executive functioning^48^. Consequently, it has been interpreted as a relatively consistent disposition reflecting the regulation of cognitive functions and situational adaptability ^[e.g.,49,50^. LF/HF ratio, on the other hand, is associated with RSA-related heart rate changes and has been suggested to reflect vagal activity and the ratio between sympathetic and parasympathetic nervous system activity^48^ which increases/decreases as a function of situational physiological arousal level/ orthostatic load, as, for example, induced by body position^51^. Of note, as we lacked the technological possibility of sending task triggers to the ECG recorder, all these heart rate measures were collapsed across a whole experimental session of approximately 45 min.

Since three out of the 72 measurement occasions were led by a different experimenter (AZ) whose device was incompatible with the HRV + app, heart rate data from these occasions were recorded with the Heart Rate Monitor app (BM Innovations GmbH). While recordings of two of these occasions were missing due to technical issues, this app only allowed the export of the average BPM values over the recording, but not the RR intervals over time – thus, we could not calculate time-domain and -frequency measures (HRV and LF/HF) for the remaining occasion. We obtained only the original BPM value for this measurement.

### Assessment of personal fitness characteristics

The short form of the International Physical Activity Questionnaire (IPAQ) asks about individual physical activity habits in three domains: *walking*, *moderate-intense* activity, and *vigorous-intense* activity^46^. IPAQ questionnaire data was processed according to the processing and analysis guidelines^52^, first weighting each activity type by its energy requirements defined in multiples of resting metabolic tasks (METs; 1 MET equals 3.5 ml/kg/min oxygen consumption) to get a MET-minutes score as a measure of activity volume (equivalent to energy expenditure in kilocalories for a 60 kg person per minute). We used the recommended MET weights for each domain (Walking = 3.3; Moderate activity = 4.0; Vigorous activity = 8.0^52^) to compute the individual domain-specific MET minutes/week before summing them up to a total physical activity MET minutes/week score per subject (PA). The average score of self-reported physical activity in our sample was 2465.26 MET-minutes/week (*SD*_PA_ = 1848.58 MET-minutes/week, *Min*_PA_ = 358 MET-minutes/week, *Max*_PA_ = 8079 MET-minutes/week).

The body mass index (BMI) was calculated from the self-reported individual height ($\:l$) and weight ($\:m$) values using the standard formula $\:BMI=\:\frac{m}{{l}^{2}}$. One participant’s BMI was missing because this person did not want to indicate their weight and height. The average BMI in our sample was 22.11 (*SD*_BMI_ = 2.74, *Min*_BMI_ = 17.78, *Max*_BMI_ = 29.73).

### Statistical analysis

Based on recently reported measurement issues with difference scores in tasks for executive functions and inhibition measures specifically^14,53^ we did not aggregate over trials within participants to calculate the traditional congruency effect. Instead, in line with other recent work^19^, we utilized hierarchical mixed effects models to account for measurement error on the trial level while also controlling for interindividual differences in response speed and accuracy between participants. Of note, before the data were entered into the models, intraindividual outlier trials were excluded in line with criteria recently applied in our lab^38^: Trials were excluded if they deviated > 3 SDs relative to the individual’s (stand/sit-)condition-specific mean RT as a subject-specific cutoff. Further, we excluded remaining fast responses that were given quicker than 150 ms, or responses slower than 3000 ms. For the Stroop task, on average, this resulted in the exclusion of 2.02% of a given subject’s trials (*SD* = 0.89%) and the removal of 1.91% of individual trials in the Navon task (*SD* = 1.09%). To stay consistent with recent analyses on the *posture x Stroop effect* interaction^19^ we ran the analyses on log-transformed RTs to account for the typical right-skewed distribution of RT data.

For our main analysis, we identified model-specific appropriate random effect structures through a stepwise process based on likelihood ratio tests using the package *buildmer*^54^. Before this, we derived a suitable fixed effect structure that allowed us to test the hypotheses while avoiding multicollinearity by keeping the variance inflation factor below acceptable cutoffs^55,56^. This model selection procedure is described in more detail in Supplementary Material 4. The final model tested the three-way interactions of *congruency x posture x PA*, and *congruency x posture x BMI*, while controlling for the direct influence of HRV, and included additional model-specific random effects to account for possible cluster-induced correlations in the data.

In equation form, the model was specified as:

$$\begin{aligned} \widehat{{y_{{ij}} }} & = \beta _{{0i}} + \beta _{{1i}} congruency_{{ij}} + \beta _{{2i}} \,posture_{{ij}} + \beta _{{3i}} hrv_{{ij}} + \beta _{{4i}} PA_{i} + \beta _{{5i}} BMI_{i} \\ & \quad + {\text{~}}\beta _{{6i}} \left( {congruency_{{ij}} \times posture_{{ij}} } \right) + \beta _{{7i}} \left( {congruency_{{ij}} \times PA_{i} } \right) \\ & \quad + \beta _{{8i}} \left( {congruency_{{ij}} \times BMI_{i} } \right) + \beta _{{9i~}} \left( {posture_{{ij}} \times PA_{i} } \right) + \beta _{{10i}} (posture_{{ij}} \\ & \quad \times BMI_{i} ) + ~\beta _{{11i}} \left( {congruency_{{ij}} \times posture_{{ij}} \times PA_{i} } \right) + \beta _{{12i}} (congruency_{{ij}} \\ & \quad \times posture_{{ij}} \times BMI_{i} ) \\ \end{aligned}$$

where $\:\widehat{{y}_{ij}}$ is the predicted dependent variable (Navon or Stroop RT or accuracy, respectively), with *i* indexing subjects and *j* indexing individual observations, $\:{\beta\:}_{0i}$ is the intercept, $\:{hrv}_{ij}\:$is the (scaled) heart rate variability measure, $\:{congruency}_{ij}$ and $\:{posture}_{ij}$ are effect-coded categorical predictors (congruent vs. incongruent, sitting vs. standing), and $\:{PA}_{i}$ and $\:{BMI}_{i}$ are the (scaled) measures of physical activity and body mass index, respectively. If not mentioned otherwise (see Supplementary Material 4), the slope parameters were fixed effects only, i.e., for example $\:{\beta\:}_{4i}\:=\:{\beta\:}_{4}$.

Linear mixed models (LMM) were run using functions from the *lme4* package in R^57^ using the BOBYQA optimizer with optimization limits of 200k iterations. Categorical predictors *congruency* (congruent = − 1; incongruent = 1) and *posture* (sit = − 1; stand = 1) were effect-coded (by applying sum contrasts) to facilitate the interpretation of the main effects and interaction effects^58–60^. Consequently, all effects can be interpreted as main effects relative to the grand average across all conditions and covariate levels, as opposed to treatment contrasts (“dummy coding”), which would allow only for a “simple effects”-interpretation relative to a pre-specified reference condition^58–60^. An alternative analysis with treatment contrasts is reported in Supplementary Material 7.

Of note, before we conducted the statistical analyses, all continuous predictor variables were rescaled (standardized by centering on the grand mean around *M* = 0 with *SD* = 1) to mitigate numerical issues in model fitting and to facilitate the interpretation of the output (For a control analysis with continuous covariates centered within posture-cluster instead of the grand mean, see Supplementary material 6). Notably, to test the effects on accuracy, for these models, we used *generalized* LMMs (GLMM) with binomial distributions and logit link functions due to the categorical nature of the dependent accuracy variable. Satterthwaite’s method was used to approximate the degrees of freedom for the LMMs, and Wald’s method was used for the GLMMs.

## Results

### Manipulation check: modulation of heart rate by posture and physical fitness

We first applied linear mixed models (including random intercepts per subject) to test if posture generally modulated cardiac activity while accounting for self-reported physical activity and individual BMI. Proving a general influence of posture manipulation on the human body, these tests revealed that relative to sitting posture, standing significantly increased BPM (*M*_standing_ = 94.29, *SD*_standing_ = 14.98; *M*_sitting_ = 82.50, *SD*_sitting_ = 13.02; df = 34.85, *t* = 5.04, *p* < .001) and LF/HF power ratio (*M*_standing_ = 5.74, *SD*_standing_ = 4.04; *M*_sitting_ = 2.93, *SD*_sitting_ = 1.81; df = 34.07, *t* = 5.89, *p* < .001) and led to lower HRV (*M*_standing_ = 51.97, *SD*_standing_ = 22.23; *M*_sitting_ = 62.35, *SD*_sitting_ = 23.67; df = 34.74, *t* = -2.49, *p* = .02). Higher BMI had a significant negative association to HRV with an estimated change of − 7.55 per unit increase in scaled BMI (df = 32.74, *t* = − 2.46, *p* = .02), whereas higher self-reported physical activity was significantly related to the individual BPM (estimated change of − 5.77 BPM per unit increase in scaled PA; df = 32.93, *t* = − 3.23, *p* < .01). For a full summary of the models related to this manipulation check, see Supplementary Material 1.1.

### Manipulation check: behavioral conflict effects

Next, for both control tasks, we tested the congruency effect for response time in correctly responded trials and for accuracy on statistical significance. Given the proposed mediation mechanism of the posture effect on cognitive control via physiological arousal, we already included HRV (our main measure of physiological arousal) and its interaction with the congruency factor to test this step of the proposed mechanism.

For a summary of these models, see Supplementary Material 1.2. The typical Stroop effect was replicated both in RT (*M*_congruent_ = 609.89 ms, *SD*_congruent_ = 269.72 ms; *M*_incongruent_ = 680.74, *SD*_incongruent_ = 309.75; df = 5534, *t* = 12.61, *p* < .001) and in task accuracy (*M*_congruent_ = 97.23% correct, *SD*_congruent_ = 16.43%; *M*_incongruent_ = 95.88% correct, *SD*_incongruent_ = 19.88%; z = − 3.08, *p* < .01).

Similarly, Navon RT (*M*_congruent_ = 616.25 ms, *SD*_congruent_ = 204.01 ms; *M*_incongruent_ = 671.26 ms, *SD*_incongruent_ = 219.78 ms; df = 3934, *t* = 14.74, *p* < .001) and accuracy (*M*_congruent_ = 92.61% correct, *SD*_congruent_ = 25.59%; *M*_incongruent_ = 87.79% correct, *SD*_incongruent_ = 32.74%; *z* = − 5.81, *p* < .001) differed significantly between congruency conditions. HRV, as our index of physiological arousal, did not show a direct effect on response times or task accuracies (all *p’s* > 0.2). Only in the two Navon-related models did the interaction effect reach significance, implying a modulation of the congruency effect depending on physiological arousal level (HRV) (Navon accuracy model: Odds Ratio = 0.61; z = − 3.92, *p* < .001; Navon log-RT model: df = 3933, t = 2.93, *p* < .01).

### Posture effects on Stroop control

Subsequently, we ran a series of linear mixed effects models for the main analysis, as detailed in the Statistical Analyses section. Regarding Stroop task results, see Table 2 for a full summary of the two models on RT and accuracy.

**Table 2:** Posture modulation of stroop congruency effects.

	Stroop log-RT		Stroop Acc.	
Predictors	Estimates	*p*	Odds ratios	*p*
(Intercept)	6.40 (6.33–6.47)	**< 0.001**	41.30 (29.92–57.00)	**< 0.001**
HRV	0.00 (− 0.04–0.04)	0.892	1.01 (0.77–1.32)	0.955
Congruency [incong.]	0.05 (0.04–0.06)	**< 0.001**	0.78 (0.67–0.91)	**0.002**
Posture [stand]	− 0.00 (− 0.03–0.02)	0.899	1.06 (0.90–1.25)	0.454
Physical Activity (PA)	− 0.01 (− 0.08–0.06)	0.785	0.96 (0.71–1.29)	0.773
BMI	0.02 (− 0.05–0.09)	0.524	0.85 (0.63–1.16)	0.313
Congruency [incong.] × Posture [stand]	0.00 (− 0.01–0.01)	0.602	0.91 (0.78–1.06)	0.231
Congruency [incong.] × PA	− 0.00 (− 0.01–0.00)	0.357	0.96 (0.83–1.11)	0.620
Posture [stand] × PA	0.02 (− 0.01–0.04)	0.120	1.07 (0.93–1.24)	0.353
Congruency [incong.] × BMI	0.00 (− 0.00–0.01)	0.485	0.98 (0.85–1.12)	0.736
Posture [stand] × BMI	0.01 (− 0.01–0.03)	0.457	0.91 (0.79–1.05)	0.184
(Congruency [incong.] × Posture [stand]) × PA	0.00 (− 0.00–0.01)	0.244	0.83 (0.72–0.96)	**0.015**
(Congruency [incong.] × Posture [stand]) × BMI	0.00 (− 0.01–0.01)	0.501	0.93 (0.81–1.07)	0.307
Random effects				
σ^2^	0.09		3.29	
τ_00_	0.04 _sb_		0.60 _sb_	
τ_11_	0.00 _sb.posture1_			
ρ_01_	0.09 _sb_			
ICC	0.35		0.15	
N	36 _sb_		36 _sb_	
Observations	5573		5760	
Marginal R^2^ / Conditional R^2^	0.027 / 0.363		0.039 / 0.187	

Categorical predictors *congruency* (congruent = − 1; incongruent = 1) and *posture* (sit = − 1; stand = 1) were effect-coded (by applying sum contrasts). Consequently, all effects can be interpreted as main effects relative to the grand average across all conditions and covariate levels (for example, coefficients for the predictor “Congruency [incong.]“ describe the main effect of incongruent trial conditions, coefficients for the predictor “Posture [stand]“ describe the main effect for the standing posture condition). All continuous predictor variables were z-standardized before inclusion into the models. Estimates for RT-models thus represent the change in log-RT when the predictor variable increases by 1 SD while holding other variables constant; Estimates of the Accuracy-models represent the change in the Odds Ratio for responding correctly when the predictor variable increases by 1 SD while holding other variables constant. Values in parentheses refer to the 95% confidence interval. Satterthwaite’s method is used to approximate the degrees of freedom for the LMMs, and Wald’s method is used for the GLMMs.

For RT, this model yielded only a significant main effect of congruency condition (as evident also in the simpler model reported above; df = 5497, *t* = 13.00, *p* < .001).

For Stroop accuracy, the main effect of congruency was again significant (*z* = − 3.11, *p* < .01). We also found a significant three-way interaction between congruency, posture, and physical activity (*z* = -2.44, *p* = .01; Fig. 3A). Thus, we ran a follow-up simple slope analysis to probe this interaction^61^ which indicated that posture modulates the Stroop congruency effect of accuracy differentially depending on a person’s physical activity level: Participants with lower physical fitness showed greater benefits from the standing condition (had a less detrimental effect of incongruent trials on task accuracy) than more physically active participants. In fact, the simple slope analysis showed that a robust congruency effect in Stroop accuracy only seemed to emerge for relatively fit people in the standing condition (Fig. 3A; *z* = − 2.98, *p* < .001 for people with average PA level in standing condition; *z* = − 3.27, *p* < .001 for people 1 SD above average PA in standing condition; all other *z* < − 1.93, all other *p* > = 0.05, including the sitting condition).

A comparable pattern was replicated when checking the result’s robustness by accounting for the LF/HF ratio instead of HRV as an alternative measure of physiological arousal (see Supplementary material 3), and when centering continuous covariates within (posture-)cluster instead of centered on the grand mean (see Supplementary material 6).

To test if we found evidence *against* an influence of posture on the Stroop RT effect (the classic effect reported in the original study by Rosenbaum et al.^45^), we exploratively ran an additional Bayesian LMM (see Supplementary 5 for detailed model specifications) using *brms* in R^62^. Posteriors were computed for each parameter using functions from the *bayestestR* package^63^. The posterior mass for the congruency x posture parameter estimate was relatively evenly split at the null (pd = 63.24%), indicating that a null effect for this interaction is highly credible. For a complete summary of the Bayesian analyses, see Supplementary material 5.

### Posture effects on Navon control

**Fig. 2:**
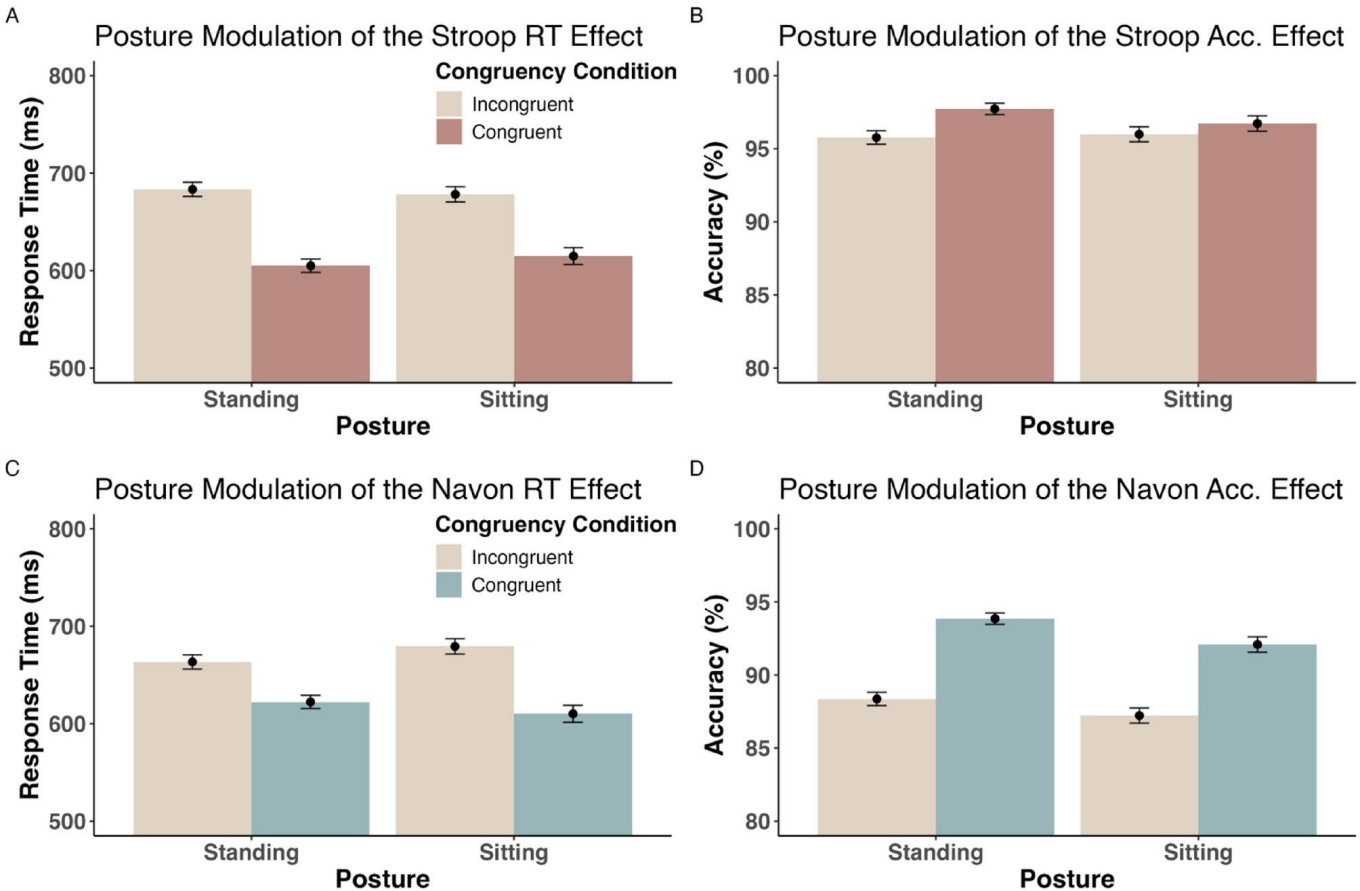
Direct posture effects on cognitive control. Bar plots illustrate the impact of posture (X-axis) and congruency condition (coloring, see legend) on response time in correctly responded trials (Y-Axis in panels A and C) and on the percentage of correctly responded trials (Accuracy; Y-Axis in panels B and D). Error bars represent the standard error of the mean.

We then ran equivalent models for the Navon task (see Table 3). Here, we again observed significant main effects of congruency condition on RT (df = 3334, *t* = 6.52, *p* < .001) and on accuracy (*z* = − 5.76, *p* < .001). Most crucially, there was a significant interaction between congruency condition and posture on RT (df = 3899, *t* = − 2.34, *p* = .02), implying a reduced (*b* = 0.04) Navon congruency effect of RT in standing posture (Fig. 2C) opposed to sitting position (*b* = 0.06; *p* < .001 in both conditions; as indicated by a simple slope analysis. Note that due to the application of sum contrasts, *b*’s refer to the effect relative to the grand average across conditions. I.e., the predicted difference between congruency conditions is 2*b).

**Table 3:** Posture modulation of Navon congruency effects.

	Navon log-RT		Navon Acc.	
Predictors	Estimates	*p*	Odds ratios	*p*
(Intercept)	6.45 (6.40–6.50)	**< 0.001**	18.58 (12.02–28.72)	**< 0.001**
HRV	− 0.01 (− 0.04–0.02)	0.609	1.11 (0.87–1.43)	0.404
Congruency [incong.]	0.05 (0.03–0.06)	**< 0.001**	0.66 (0.52–0.85)	**0.001**
Posture [stand]	− 0.01 (− 0.03–0.02)	0.680	1.16 (1.02–1.31)	**0.021**
Physical Activity (PA)	− 0.04 (− 0.08–0.00)	0.058	1.05 (0.68–1.62)	0.811
BMI	0.06 (− 0.07–0.19)	0.317	0.96 (0.62–1.48)	0.842
Congruency [incong.] × Posture [stand]	− 0.01 (− 0.01 – − 0.00)	**0.019**	0.96 (0.86–1.07)	0.432
Congruency [incong.] × PA	− 0.00 (− 0.02–0.01)	0.650	1.26 (1.00–1.58)	**0.046**
Posture [stand] × PA	0.01 (− 0.01–0.04)	0.236	0.95 (0.85–1.06)	0.368
Congruency [incong.] × BMI	0.00 (− 0.01–0.02)	0.758	1.03 (0.82–1.30)	0.779
Posture [stand] × BMI	− 0.00 (− 0.03–0.02)	0.892	1.02 (0.91–1.15)	0.704
(Congruency [incong.] × Posture [stand]) × PA	0.00 (− 0.00–0.01)	0.202	0.99 (0.89–1.11)	0.908
(Congruency [incong.] × Posture [stand]) × BMI	− 0.00 (− 0.01–0.00)	0.423	0.96 (0.85–1.08)	0.456
Random Effects				
σ^2^	0.04		3.29	
τ_00_	0.01 _sb_		1.47 _sb_	
τ_11_	0.00 _sb.posture1_			
	0.00 _sb.congruent1_		0.27 _sb.congruent1_	
	0.08 _sb.bmi_scaled_			
ρ_01_	− 0.03		− 0.18 _sb_	
	0.37			
	0.66			
ICC	0.73		0.35	
N	36 _sb_		36 _sb_	
Observations	3972		4395	
Marginal R^2^ / Conditional R^2^	0.059 / 0.746		0.049 / 0.379	

Categorical predictors *congruency* (congruent = − 1; incongruent = 1) and *posture* (sit = − 1; stand = 1) were effect-coded (by applying sum contrasts). Consequently, all effects can be interpreted as main effects relative to the grand average across all conditions and covariate levels (for example, coefficients for the predictor “Congruency [incong.]“ describe the main effect of incongruent trial conditions, coefficients for the predictor “Posture [stand]“ describe the main effect for the standing posture condition). All continuous predictor variables were z-standardized before inclusion into the models. Estimates for RT-models thus represent the change in log-RT when the predictor variable increases by 1 SD while holding other variables constant; Estimates of the Accuracy-models represent the change in the Odds Ratio for responding correctly when the predictor variable increases by 1 SD while holding other variables constant. Values in parentheses refer to the 95% confidence interval. Satterthwaite’s method is used to approximate the degrees of freedom for the LMMs, and Wald’s method is used for the GLMMs.

**Fig. 3:**
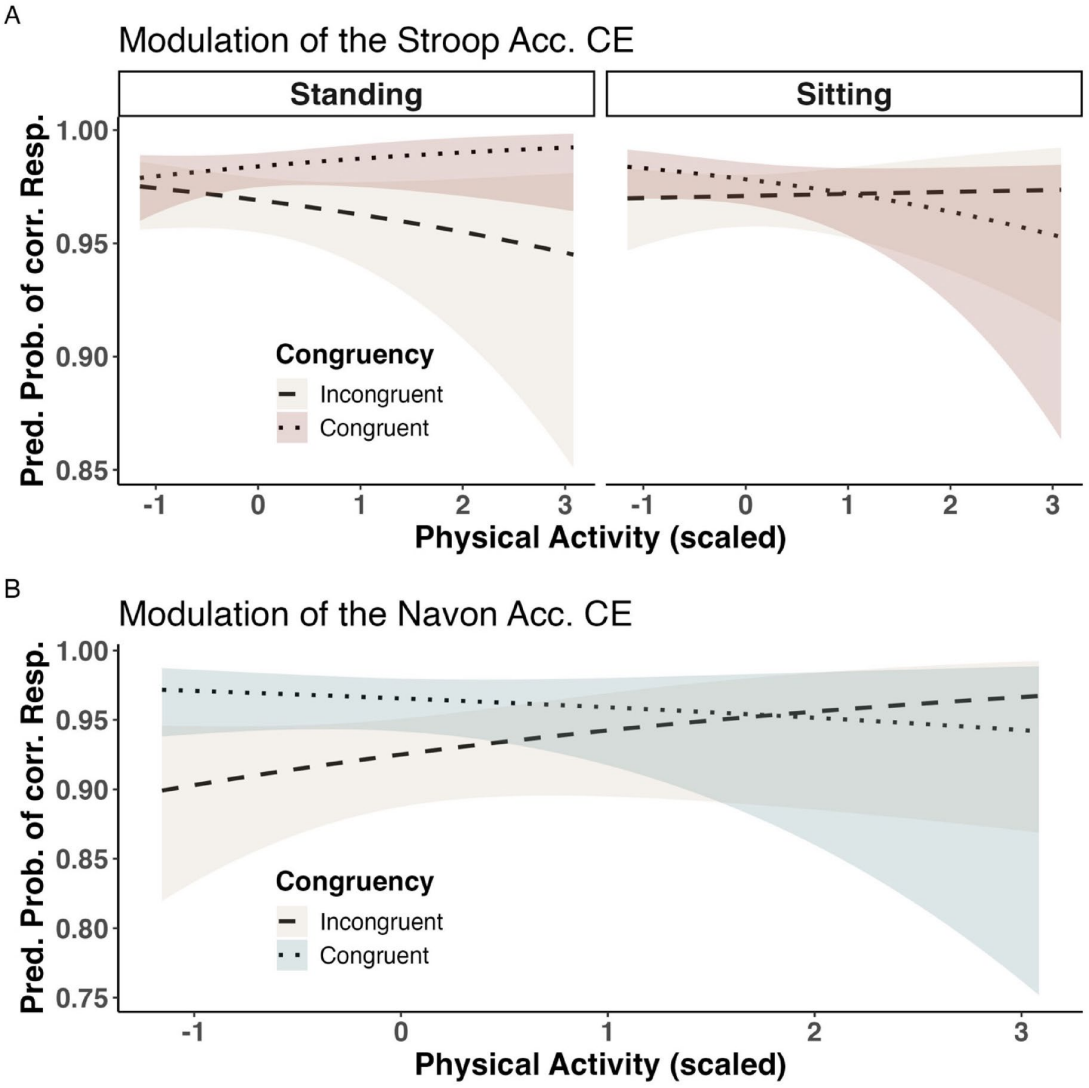
Physical fitness as influence on the posture effects and congruency effects. The plots illustrate how physical fitness level (X-Axis, scaled) modulates the effects of posture (different plot facets in panel A) and congruency condition (line type in panels A and B, see legend) on the predicted probability to respond correctly (Accuracy; Y-Axis). Acc. = accuracy, CE = congruency effect.

The analysis further yielded a significant main effect of standing posture on overall Navon accuracy (*z* = 2.31, *p* = .02; Fig. 2D). Physical activity also significantly modulated the congruency effect on Navon accuracy (*z* = 2.72, *p* < .01; Fig. 3B). The follow-up simple slope analysis revealed that physically fitter (+ 1 SD) individuals had a less detrimental effect of incongruent trials (b = − 0.18, z = − 1.01, *p* = .31) than people with average (b = -0.41, z = − 3.22, *p* < .001) or low (− 1 SD) self-reported fitness levels (b = -0.64, z = − 3.77, *p* < .001).

Control analyses accounting for the LF/HF ratio instead of HRV as an alternative measure of physiological arousal yielded a virtually similar pattern of results (see Supplementary material 3). Similarly, comparable results were observed when centering continuous covariates within (posture-)cluster instead of centered on the grand mean (see Supplementary material 6).

We also ran an additional exploratory Bayesian analysis on Navon RT (where we found the significant *posture x congruency* interaction of interest), equivalent to that on Stroop RT reported above (see Supplementary material 5). Here, nearly all (pd > 97%) of the posterior mass pointed towards a negative estimate of the interaction effect, consistent with the significant effect in the frequentist analysis. This effect remained credible also in a series of prior sensitivity checks^64^with different SD values (0.5, 0.01, 0.001, 0.0001) for the Gaussian prior for the interaction parameter around zero (SD = 0.2 as default for main analysis, see Supplementary material 5). Across all prior settings, and even in the most conservative prior setting (most peaked at the null; SD = 0.0001), these checks demonstrated highly credible effect estimates for the posture x congruency interaction (pd > 97%). Although numerically small, the estimated effect size refers to a considerable 10% decrease (− 6 ms) in the congruency effect (60 ms) in standing position.

### Mediation analysis

Since we found a significant modulation of the Navon RT congruency effect by standing posture in our main analysis, we then aimed to clarify whether this effect was mediated by changes in physiological arousal as indexed by HRV. After having replicated the direct effect of posture on the proposed mediator HRV and having established the direct effect of HRV on the Navon RT congruency effect (see above), we tested the relationship between posture, HRV, and the congruency effect more rigorously in three simpler models with comparable by-participant intercepts and removing the non-significant effects of BMI and physical activity (Table 4).

Due to the data structure (each participant had multiple observations of reaction time per posture and congruency condition, but only two HRV measurements - one per posture), we were not able to run standard mediation tests because the mediator and outcome were not measured on the same level of our nested data. Instead, we manually computed the indirect effect using the coefficients from the models and tested its significance via bootstrapping. For this, we extracted the coefficient for the effect of posture on HRV from the respective model (*b*_posture−> HRV_ = − 0.22; see Table 4, middle model columns) as well as the coefficient for the effect of HRV on the congruency effect of Navon log-RT (*b*_HRVxCongruency−> NavRT_ = 0.01; see Table 4, right model columns). The indirect effect was then manually calculated as the product of both coefficients; *Indirect Effect* = *b*_posture−> HRV_ * *b*_HRVxCongruency−> NavRT_. Next, the significance of the indirect effect was tested via bootstrapping using *boot* in R^65,66^ resampling the data 1000 times, refitting the models, and calculating the distribution of the indirect effect across samples. The bootstrapped confidence interval calculation yielded a relatively small but statistically significant indirect effect of -0.0019 on the congruency effect of Navon log-RT (95% CI: − 0.0032, − 0.0005; based on 1000 bootstrap replicates), suggesting a full mediation of the posture effect on the congruency effect of Navon log-RT through HRV, given that the direct effect of posture became non-significant after accounting for the influence of HRV (Table 4). Although still small, again note that due to the application of sum contrasts, *b*’s refer to the effect relative to the grand average across conditions, i.e., the predicted *difference between conditions* is 2*b.

**Table 4:** Models testing the total effect and indirect effect of posture on the congruency effect of Navon RT.

	Navon log-RT		HRV		Navon log-RT				
Predictors	Estimates	*p*	Estimates	*p*	Estimates			*p*	
(Intercept)	6.44 (6.37–6.50)	**< 0.001**	− 0.00 (− 0.29–0.28)	0.983	6.44 (6.38–6.50)			**< 0.001**	
Congruency [incong.]	0.05 (0.04–0.06)	**< 0.001**			0.05 (0.04–0.06)			**< 0.001**	
Posture [stand]	− 0.00 (− 0.01–0.00)	0.190	− 0.22 (− 0.40 – − 0.04)	**0.018**	− 0.00 (− 0.01–0.00)			0.203	
Congruency [incong.] × Posture [stand]	− 0.01 (− 0.01 – − 0.00)	**0.043**			− 0.01 (− 0.01–0.00)			0.125	
HRV					0.00 (− 0.01–0.02)			0.471	
Congruency [incong.] × HRV					0.01 (0.00–0.02)			**0.011**	
**Random Effects**									
σ^2^	0.04		0.53		0.04				
τ_00_	0.03 _sb_		0.43 _sb_		0.03 _sb_				
ICC	0.42		0.44		0.42				
N	36 _sb_		36 _sb_		36 _sb_				
Observations	4085		70		3972				
Marginal R^2^ / Conditional R^2^	0.031 / 0.442		0.048 / 0.471		0.033 / 0.443				

Categorical predictors *congruency* (congruent = − 1; incongruent = 1) and *posture* (sit = − 1; stand = 1) were effect-coded (by applying sum contrasts). Consequently, all effects can be interpreted as main effects relative to the grand average across all conditions and covariate levels (for example, coefficients for the predictor “Congruency [incong.]“ describe the main effect of incongruent trial conditions, coefficients for the predictor “Posture [stand]“ describe the main effect for the standing posture condition). All continuous predictor variables were z-standardized before inclusion into the models. Values in parentheses refer to the 95% confidence interval. Satterthwaite’s method is used to approximate the degrees of freedom.

## Discussion

The present study is the first to rigorously test the *posture x cognitive control* interaction under the control of individual fitness measures, such as BMI, physical activity, and posture-dependent heart rate measures, that may influence the attentional demand an individual experiences from posture manipulations. Importantly, we also extend former findings by adding a second measure of cognitive control (Navon task) that assesses qualitatively different and previously ignored (space-based) aspects of cognitive control.

Whereas seminal work had found that the Stroop interference effect is reduced in standing position^6,15^ these findings were questioned by subsequent replication attempts which failed to reproduce the same results^19,29^. This inconsistency in the findings regarding the *Stroop x posture* interaction has previously been suggested to be due to subtle factors, such as sample characteristics of the participant populations under investigation, that have not yet been properly controlled for but may lead to individuals being differentially affected by posture manipulations^29^. After controlling for such characteristics as BMI and physical fitness for the first time, the present work again demonstrates evidence *against* an effect of posture on the Stroop interference effect (Fig. 2A and B). This aligns with other works reporting no significant influence of posture on Stroop control^5,28–30^ or evidence against such an effect^19^. Importantly, we show that the (non-)existence of the posture effect on Stroop RT control is independent of an individual’s fitness in terms of BMI and weekly exercise level. One possible explanation would be that the increase in arousal and attentional demands is insufficient to have a reliable effect on Stroop RT control in standing posture. For example, a recent study by Peskar and colleagues^67^ showed that only walking (compared to standing and sitting posture) tended to facilitate faster interference suppression, accompanied by a change in control-related frontocentral EEG signatures. It may be that – contrary to the reversed U-curve relation proposed by Smith et al.^6^ – simple standing may not generally represent the optimal arousal level to facilitate mechanisms involved in Stroop interference control. However, what’s unique about more demanding postural situations to facilitate (instead of decrease) cognitive performance remains to be tested, since other works have shown detrimental effects in such contexts^10,26^. Moreover, when considering the congruency effect of Stroop *accuracy* instead of RT, posture did significantly modulate cognitive control, but only for relatively fit people, and inducing, however, a *higher* CE of accuracy (lower cognitive control) in standing position. Consequently, in sum, we find no signs of enhanced Stroop control during standing posture – if anything, standing seems to *impair* interference control regarding task accuracy for a subgroup of people with regular physical training. This was notably the only case (across tasks) where physical fitness measures modulated the posture effect on congruency: Generally, posture effects on cognitive control thus seem largely independent of sample characteristics like BMI or physical training across tasks and dependent variables.

In contrast to the overall null effect of standing posture on Stroop RT control, in the Navon task, we did find a significant posture effect on the RT congruency effect (Fig. 2C), implying facilitated spatial conflict processing in standing posture, fully mediated by enhanced physiological arousal as measured in reduced HRV. This further corroborates the notion that postural changes may differentially influence diverse aspects of cognitive control: Whereas feature-based (RT-) control in the Stroop task overall seems to be relatively unaffected by adopting a standing posture, just as flanker inhibition^4,41,42^ we observe that space-based perceptual control in the Navon task benefits from the enhanced physiological arousal in standing position. This further demonstrates that established cognitive control tasks assess substantial task-specific control mechanisms and seem far from measuring a unitary construct^14,37^. As suggested before^38^ particularly the Stroop and Navon tasks, which were utilized in the present work, may represent qualitatively different inhibitory control mechanisms: feature-based (Stroop) vs. space-based (Navon) control^43,44^.

Considerable differences in the two tasks regarding the involved cognitive control processes are also supported by neuroimaging studies: For the Navon task, a strong involvement of the inferior frontal gyrus has previously been demonstrated, which is engaged in inhibition of attention and shows increased activation in incongruent as opposed to congruent Navon trials^68^. Furthermore, Navon-related activations were demonstrated in other brain regions associated with perceptual integration (anterior superior temporal gyrus), attention and visual processing (inferior/middle occipital gyrus, lingual gyrus), and conflict monitoring (medial frontal gyrus/anterior cingulate, middle ﻿﻿frontal gyrus^68^). Stroop task performance, on the other hand, is associated with activation of the inferior frontal junction^69^ the anterior cingulate cortex^69–72^the pre-supplementary motor area^69,72^ frontopolar structures such as the superior frontal gyrus^69–71^ orbitofrontal and parietal structures^70–72^thalamus, and lingual gyrus^71^and the insula^69^. Even though partially overlapping (e.g., frontopolar structures, anterior cingulate cortex ^cf. 73^), the involvement of other task-specific brain areas might suggest that, indeed, different forms of attention are involved in solving interference in these two tasks. This idea is further corroborated by a recent study that examined subgraphs of functional brain networks, revealing dynamic changes in both similar and distinct brain regions during the execution of Navon and Stroop tasks^39^. Thus, the differential effects of posture on Stroop and Navon performance might be attributed to the engagement of task-specific visuo-spatial attentional abilities and the distinct brain networks activated for each task. The fact that we do find posture modulations of space-based as opposed to color-based control also aligns with previous work that has shown enhanced visual attention and visual search performance in standing positions^5–7^. However, it remains curious why other researchers could not find posture effects on cognitive control in flanker paradigms^4,41,42^ which are arguably also rather space-based than feature-based. It may be interesting for future work to disentangle the task-specific mechanisms that lead to such differential effects.

Most importantly, we show that the posture effect on cognitive control in the Navon task is fully mediated by physiological arousal measured in HRV, indexing the activation and balance of (para-)sympathetic systems^48^. To our knowledge, this is the first support for the theorized mechanism that it may be the increased demands for postural control during standing that enable enhanced selective attention^15^. Due to the load that is induced through additional postural control and which is reflected in higher physiological arousal, distractor interference may be diminished^6,15,19,20,26^. This also aligns with other findings demonstrating that sleep quality-dependent (supine-) posture effects on N-Back performance may partly be explained by HRV parameters^74^. It further supports Load Theory, as our more resource-intensive standing posture manipulations allowed less processing of distracting information and thus diminished the interference of spatial distractors ^20,see also 16^. However, it is important to note that this mediation mechanism needs further verification (or falsification) by future studies. While we found the posture effect to be mediated by physiological arousal, our experimental design does not allow unequivocal inferences about a *causal* mechanism through physiological arousal since we had to aggregate cardiac measures across the testing session. Thus, at present, it is not entirely clear if the arousal increase precedes or follows the enhancement in cognitive control in standing position. If the physiological arousal mechanism is validated, it could be that this posture-induced mechanism unfolds through cardio-afferent body-brain interactions, since HRV reflects the relative durations of the systolic and diastolic cardiac cycle phases. Cardio-afferent traffic functions via arterial baroreceptors and has been shown to modulate perceptual processing and inhibitory control depending on phases of the cardiac cycle (systole vs. diastole) across various tasks, such as the Simon^75^stop signal^76^and Go/No-Go tasks^77,78^. The current study did not link cardiac measurements to specific trial conditions during online measurement, but future research could offer deeper insights by examining posture-induced effects on heart signals at the trial level. Our finding also aligns with a body of literature demonstrating that both dispositional and situational measures of HRV correlate with executive functioning^48,50,79,80^ and with inhibitory control efficiency specifically^76,81^supposedly through neurovisceral integration (for more details, see^50,82^).

Apart from these differential influences on the congruency effects, it is noteworthy that we do find that standing posture increased *overall* accuracy in the Navon task. This questions findings of increased error rates in standing conditions^5,6^but is in line with other previous reports of enhanced overall accuracy in cognitive control tasks in the upright position^27^. It may be due to increased alertness in standing versus sitting position, as proposed in earlier work: In addition to the increased physiological arousal, it has been hypothesized that standing posture may be associated with mental states (e.g., fight or flight) that are accompanied by increased alertness and facilitated attentional processing^6^, which may have helped with effective spatial filtering during Navon task performance. This is also corroborated by recent evidence that perceived task effort and mental fatigue are reduced during standing posture^42^.

Some aspects of the current work may be seen as limitations to our conclusions. While we were the first, to our knowledge, who accounted for individual fitness characteristics to clarify previously mixed findings, our analyses were limited to a student sample. Even though our sample was still relatively diverse in these characteristics, with a broad spectrum of self-reported physical activity levels (*M*_PA_ = 2465.26 MET-minutes/week, *SD*_PA_ = 1848.58 MET-minutes/week, *Min*_PA_ = 358 MET-minutes/week, *Max*_PA_ = 8079 MET-minutes/week) and including participants traditionally classified as underweight (*Min*_BMI_ = 17.78 < 18.5) and as overweight (*Max*_BMI_ = 29.73 > 25), even close to obesity (BMI ≥ 30), the fact of it being a student sample naturally constrained the variability in such individual characteristics and may have limited our potential to find moderating influences on the posture effect. Future studies could aim for a broader sample, including elderly or obese people, to replicate the present findings.

## Conclusion

We demonstrate that feature-based and space-based cognitive control are differentially affected by postural demands, likely through the mechanism of enhanced physiological arousal. While shedding more light on the influence of bodily states on cognitive functioning, our results also further question the unity of the construct of cognitive control^14,37^ and its physiological underpinnings. Especially given the overall trend of increased sitting time in human societies^1,83^ our findings may have important implications for standing-related ergonomics and workplace environments in which individuals rely on efficient spatial-attentional filtering.

## Electronic supplementary material

Below is the link to the electronic supplementary material.

Supplementary Material 1

## Data Availability

Experimental Material, Supplementary Material, data, and analysis scripts are available on OSF: https://osf.io/bafhd/.
