# Supplementary material for: Withstand control: standing posture differentially affects space-based and feature-based cognitive control through enhanced physiological arousal
Source: Sci Rep. 2025 Jul 20;15:26347. doi: 10.1038/s41598-025-11692-6 (PMC12277459; doi:10.1038/s41598-025-11692-6)
Supplement: Supplementary file 1 — Supplementary Material 1 [file 41598_2025_11692_MOESM1_ESM.pdf]

Supplementary Material

1. MANIPULATION CHECKS......2
1.1 Modulation of Heart Rate by Posture and Physical Fitness......2
1.2 Behavioral Congruency Effects......3
2. CONTROL ANALYSES WITH SUBJECT EXCLUSION......5
3. ACCOUNTING FOR ALTERNATIVE PHYSIOLOGICAL AROUSAL MEASURES......7
4. SELECTION PROCESS OF THE LMM MODEL STRUCTURE......12
5. EXPLORATORY BAYESIAN ANALYSIS OF STROOP AND NAVON RT......15
6. CONTROL ANALYSES WITH CONTINUOUS COVARIATES CENTERED WITHIN-CLUSTER.20
7. CONTROL ANALYSES WITH TREATMENT CONTRASTS......25

1. Manipulation Checks

1.1 Modulation of Heart Rate by Posture and Physical Fitness

	BPM		HRV		LF/HF	
Predictors	*Estimates*	*p*	*Estimates*	*p*	*Estimates*	*p*
(Intercept)	88.34<br>(84.64 – 92.04)	**<0.001**	57.10<br>(50.85 – 63.35)	**<0.001**	4.30<br>(3.33 – 5.28)	**<0.001**
Posture (stand)	5.85<br>(3.49 – 8.20)	**<0.001**	-5.11<br>(-9.28 – -0.95)	**0.018**	1.40<br>(0.92 – 1.89)	**<0.001**
Physical activity (PA)	-5.90<br>(-9.62 – -2.18)	**0.003**	2.73<br>(-3.54 – 9.01)	0.382	0.11<br>(-0.87 – 1.09)	0.826
BMI	1.51<br>(-2.21 – 5.22)	0.416	-7.55<br>(-13.80 – -1.31)	**0.019**	-0.27<br>(-1.24 – 0.71)	0.582
**Random Effects**						
σ^2	95.05		291.16		3.90	
τ_{00}	70.79 $_{sb}$		188.28 $_{sb}$		6.21 $_{sb}$	
ICC	0.43		0.39		0.61	
N	36 $_{sb}$		36 $_{sb}$		36 $_{sb}$	
Observations	71		70		70	
Marginal R^2 / Conditional R^2	0.302 / 0.600		0.160 / 0.490		0.170 / 0.680	

Note. All continuous predictor variables were z-standardized before inclusion into the models. Values in parentheses refer to the 95% confidence interval. Satterthwaite's method is used to approximate the degrees of freedom.

1.2 Behavioral Congruency Effects

	Stroop log-RT		Stroop Acc.		Navon log-RT		Navon Acc.	
Predictors	*Estimates*	*p*	*Odds Ratios*	*p*	*Estimates*	*p*	*Odds Ratios*	*p*
(Intercept)	6.40 (6.33 – 6.47)	**<0.001**	40.22 (29.06 – 55.66)	**<0.001**	6.44 (6.38 – 6.50)	**<0.001**	16.36 (10.76 – 24.87)	**<0.001**
Congruency [incong.]	0.05 (0.04 – 0.06)	**<0.001**	0.79 (0.68 – 0.92)	**0.002**	0.05 (0.04 – 0.06)	**<0.001**	0.72 (0.65 – 0.81)	**<0.001**
HRV	-0.00 (-0.02 – 0.01)	0.514	0.99 (0.78 – 1.27)	0.949	0.01 (-0.00 – 0.02)	0.202	1.07 (0.86 – 1.33)	0.527
Congruency [incong.] × HRV	0.00 (-0.00 – 0.01)	0.333	1.14 (0.98 – 1.33)	0.100	0.01 (0.00 – 0.02)	**0.003**	0.78 (0.69 – 0.88)	**<0.001**
**Random Effects**								
σ^2	0.09		3.29		0.04		3.29	
τ_{00}	0.04 $_{sb}$		0.63 $_{sb}$		0.03 $_{sb}$		1.40 $_{sb}$	
ICC	0.30		0.16		0.43		0.30	
N	36 $_{sb}$		36 $_{sb}$		36 $_{sb}$		36 $_{sb}$	
Observations	5573		5760		3972		4395	
Marginal R^2 / Conditional R^2	0.020 / 0.317		0.018 / 0.176		0.032 / 0.444		0.035 / 0.324	

Note. All continuous predictor variables were z-standardised before inclusion into the models. Estimates for RT-models thus represent the change in log-RT when the predictor variable increases by 1 SD while holding other variables constant; Estimates of the Accuracy-models

represent the change in the Odds Ratio for responding correctly when the predictor variable increases by 1 SD while holding other variables constant. Values in parentheses refer to the 95% confidence interval. Satterthwaite’s method is used to approximate the degrees of freedom for the LMM’s, Wald’s method is used for the GLMM’s.

2. Control analyses with subject exclusion

Here, we report control analyses excluding subjects that exceeded the cutoff of poor task performance recently applied in our lab (>20% error rate; Busch et al., 2024). No participant exceeded this cutoff in the Stroop task. Four individuals had lower than 80% accuracy for the Navon task and were thus excluded from the below-reported control analysis.

Posture Modulation of Navon Congruency Effects

	Navon log-RT		Navon Acc.	
Predictors	*Estimates*	*p*	*Odds Ratios*	*p*
(Intercept)	6.43 (6.37 – 6.49)	**<0.001**	24.03 (16.10 – 35.87)	**<0.001**
HRV	-0.03 (-0.06 – 0.01)	0.096	1.21 (0.93 – 1.57)	0.149
Congruency [incong.]	0.05 (0.03 – 0.07)	**<0.001**	0.67 (0.51 – 0.88)	**0.004**
Posture [stand]	-0.01 (-0.04 – 0.01)	0.285	1.21 (1.05 – 1.40)	**0.010**
Physical Activity (PA)	-0.00 (-0.04 – 0.04)	0.882	1.17 (0.78 – 1.76)	0.441
BMI	0.08 (-0.04 – 0.19)	0.189	1.03 (0.70 – 1.52)	0.881
Congruency [incong.] × Posture [stand]	-0.01 (-0.01 – -0.00)	**0.008**	1.02 (0.89 – 1.16)	0.780
Congruency [incong.] × PA	0.00 (-0.02 – 0.02)	0.881	1.31 (1.00 – 1.70)	**0.048**
Posture [stand] × PA	-0.00 (-0.03 – 0.02)	0.844	1.03 (0.88 – 1.20)	0.734
Congruency [incong.] × BMI	0.00 (-0.01 – 0.02)	0.580	1.02 (0.80 – 1.31)	0.850
Posture [stand] × BMI	-0.01 (-0.03 – 0.02)	0.548	1.06 (0.93 – 1.21)	0.369

(Congruency [incong.] × Posture [stand]) × PA	0.00 (-0.00 – 0.01)	0.307	1.01 (0.87 – 1.17)	0.898
(Congruency [incong.] × Posture [stand]) × BMI	-0.00 (-0.01 – 0.00)	0.281	0.98 (0.86 – 1.11)	0.732
**Random Effects**				
σ^2	0.03		3.29	
τ_{00}	0.02 $_{sb}$		0.98 $_{sb}$	
τ_{11}	0.00 $_{sb.posture1}$			
	0.00 $_{sb.congruent1}$		0.29 $_{sb.congruent1}$	
	0.06 $_{sb.bmi\_scaled}$			
ρ_{01}	-0.18		-0.50 $_{sb}$	
	0.52			
	0.84			
ICC	0.71		0.28	
N	32		32 $_{sb}$	
Observations	3624		3891	
Marginal R^2 / Conditional R^2	0.088 / 0.740		0.066 / 0.326	

Note. Categorical predictors *congruency* (congruent = -1; incongruent = 1) and *posture* (sit = -1; stand = 1) were effect-coded (by applying sum contrasts). Consequently, all effects can be interpreted as main effects relative to the grand average across all conditions and covariate levels (for example, coefficients for the predictor “Congruency [incong.]“ describe the main effect of incongruent trial conditions, coefficients for the predictor “Posture [stand]“ describe the main effect for the standing posture condition). All continuous predictor variables were z-standardized before inclusion into the models. Estimates for RT-models thus represent the change in log-RT when the predictor variable increases by 1 SD while holding other variables constant; Estimates of the Accuracy-models represent the change in the Odds Ratio for responding correctly when the predictor variable increases by 1 SD while holding other variables constant. Values in parentheses refer to the 95% confidence interval. Satterthwaite’s method is used to approximate the degrees of freedom for the LMMs, and Wald’s method is used for the GLMMs.

3. Accounting for alternative physiological arousal measures

Alternative to heart rate variability (HRV), some people may argue that the ratio of low and high-frequency power in the heart rate signal (LF/HF, methods section in main text) may be a more suitable measure of physiological arousal. It has been suggested to reflect vagal activity and the ratio between sympathetic and parasympathetic nervous system activity (Shaffer & Ginsberg, 2017) and is modulated as a function of situational physiological arousal level/ orthostatic load, as, for example, induced by body position (Perini & Veicsteinas, 2003). Thus, here we report a control analysis accounting for LF/HF instead of HRV as a physiological arousal measure.

Table 3.1

Posture Modulation of Stroop Congruency Effects accounting for LF/HF

	**Stroop log-RT**		**Stroop Acc.**	
Predictors	*Estimates*	*p*	*Odds Ratios*	*p*
(Intercept)	6.40<br>(6.33 – 6.47)	**<0.001**	41.30<br>(29.89 – 57.07)	**<0.001**
HRV	0.02<br>(-0.03 – 0.08)	0.370	0.88<br>(0.66 – 1.17)	0.384
Congruency [incong.]	0.05<br>(0.04 – 0.06)	**<0.001**	0.78<br>(0.67 – 0.91)	**0.002**
Posture [stand]	-0.01<br>(-0.04 – 0.02)	0.462	1.11<br>(0.92 – 1.35)	0.264
Physical Activity (PA)	-0.01<br>(-0.08 – 0.06)	0.772	0.97<br>(0.71 – 1.31)	0.831
BMI	0.02<br>(-0.05 – 0.09)	0.492	0.85<br>(0.63 – 1.14)	0.272
Congruency [incong.] × Posture [stand]	0.00<br>(-0.01 – 0.01)	0.602	0.91<br>(0.78 – 1.06)	0.231
Congruency [incong.] × PA	-0.00<br>(-0.01 – 0.00)	0.357	0.96<br>(0.83 – 1.11)	0.625
Posture [stand] × PA	0.02<br>(-0.01 – 0.04)	0.179	1.09<br>(0.94 – 1.28)	0.254
Congruency [incong.] × BMI	0.00<br>(-0.00 – 0.01)	0.485	0.98<br>(0.85 – 1.12)	0.755
Posture [stand] × BMI	0.01<br>(-0.01 – 0.03)	0.372	0.91<br>(0.79 – 1.04)	0.166
(Congruency [incong.] × Posture [stand]) × PA	0.00<br>(-0.00 – 0.01)	0.243	0.83<br>(0.72 – 0.96)	**0.015**
(Congruency [incong.] × Posture [stand]) × BMI	0.00<br>(-0.01 – 0.01)	0.500	0.93<br>(0.81 – 1.07)	0.309
**Random Effects**				
σ^2	0.09		3.29	
τ_{00}	0.04		0.61	
τ_{11}	0.00			

ρ_{01}	0.09	
ICC	0.34	0.16
N	36	36
Observations	5573	5760
Marginal R^2 / Conditional R^2	0.030 / 0.357	0.041 / 0.191

Note. Categorical predictors *congruency* (congruent = -1; incongruent = 1) and *posture* (sit = -1; stand = 1) were effect-coded (by applying sum contrasts). Consequently, all effects can be interpreted as main effects relative to the grand average across all conditions and covariate levels (for example, coefficients for the predictor "Congruency [incong.]" describe the main effect of incongruent trial conditions, coefficients for the predictor "Posture [stand]" describe the main effect for the standing posture condition). All continuous predictor variables were z-standardized before inclusion into the models. Estimates for RT-models thus represent the change in log-RT when the predictor variable increases by 1 SD while holding other variables constant; Estimates of the Accuracy-models represent the change in the Odds Ratio for responding correctly when the predictor variable increases by 1 SD while holding other variables constant. Values in parentheses refer to the 95% confidence interval. Satterthwaite's method is used to approximate the degrees of freedom for the LMMs, and Wald's method is used for the GLMMs.

Table 3.2

Posture Modulation of Navon Congruency Effects accounting for LF/HF

	Navon log-RT		Navon Acc.	
Predictors	*Estimates*	*p*	*Odds Ratios*	*p*
(Intercept)	6.46<br>(6.40 – 6.51)	**<0.001**	18.58<br>(11.99 – 28.79)	**<0.001**
HRV	0.03<br>(-0.01 – 0.06)	0.153	0.93<br>(0.69 – 1.24)	0.602
Congruency [incong.]	0.05<br>(0.03 – 0.06)	**<0.001**	0.66<br>(0.51 – 0.84)	**0.001**
Posture [stand]	-0.01<br>(-0.04 – 0.01)	0.310	1.17<br>(0.99 – 1.37)	0.062
Physical Activity (PA)	-0.04<br>(-0.08 – 0.00)	0.057	1.07<br>(0.70 – 1.65)	0.754
BMI	0.07<br>(-0.06 – 0.20)	0.297	0.92<br>(0.60 – 1.43)	0.721
Congruency [incong.] × Posture [stand]	-0.01<br>(-0.01 – -0.00)	**0.019**	0.96<br>(0.86 – 1.07)	0.450
Congruency [incong.] × PA	-0.00<br>(-0.02 – 0.01)	0.650	1.26<br>(1.00 – 1.58)	**0.048**
Posture [stand] × PA	0.01<br>(-0.01 – 0.04)	0.313	0.97<br>(0.86 – 1.10)	0.633
Congruency [incong.] × BMI	0.00<br>(-0.01 – 0.02)	0.739	1.03<br>(0.82 – 1.31)	0.782
Posture [stand] × BMI	0.00<br>(-0.02 – 0.02)	0.913	1.03<br>(0.91 – 1.16)	0.646
(Congruency [incong.] × Posture [stand]) × PA	0.00<br>(-0.00 – 0.01)	0.201	1.00<br>(0.89 – 1.11)	0.956
(Congruency [incong.] × Posture [stand]) × BMI	-0.00<br>(-0.01 – 0.00)	0.424	0.95<br>(0.85 – 1.08)	0.441
**Random Effects**				
σ^2	0.04		3.29	
τ_{00}	0.01 $_{sb}$		1.49 $_{sb}$	
τ_{11}	0.00 $_{sb.posture1}$			

	0.00 sb.congruent1	0.28 sb.congruent1
	0.08 sb.bmi_scaled	
ρ_{01}	-0.09	-0.22 sb
	0.35	
	0.69	
ICC	0.74	0.35
N	36 sb	36 sb
Observations	3972	4395
Marginal R^2 / Conditional R^2	0.058 / 0.752	0.049 / 0.381

Note. Categorical predictors *congruency* (congruent = -1; incongruent = 1) and *posture* (sit = -1; stand = 1) were effect-coded (by applying sum contrasts). Consequently, all effects can be interpreted as main effects relative to the grand average across all conditions and covariate levels (for example, coefficients for the predictor "Congruency [incong.]" describe the main effect of incongruent trial conditions, coefficients for the predictor "Posture [stand]" describe the main effect for the standing posture condition). All continuous predictor variables were z-standardized before inclusion into the models. Estimates for RT-models thus represent the change in log-RT when the predictor variable increases by 1 SD while holding other variables constant; Estimates of the Accuracy-models represent the change in the Odds Ratio for responding correctly when the predictor variable increases by 1 SD while holding other variables constant. Values in parentheses refer to the 95% confidence interval. Satterthwaite's method is used to approximate the degrees of freedom for the LMMs, and Wald's method is used for the GLMMs.

4. Selection process of the LMM model structure

We selected the most appropriate fixed effects and random effects structure for our main analysis as follows.

First, we checked for all four dependent variables if we could fit an LMM including all three three-way interactions of theoretical interest (congruency x posture x HRV; congruency x posture x BMI, congruency x posture x PA) without running into troubles with multicollinearity. For these checks, we worked with LMMs (or for accuracy with *generalized* LMMs (GLMM) with binomial distribution and a logit link function due to the categorical nature of the variable), including only by-participant random intercepts (see below for the selection of the random effects structure for the actual main analyses). Indeed, this fixed effect structure led to substantial multicollinearity, particularly in the two GLMM models predicting task accuracy. For example, in the Navon accuracy model, the three-way interaction of congruency x posture x HRV had variance inflation factors (VIF) > 5, and the Stroop accuracy model yielded similarly high VIF > 5 and failed to converge, implying yet too complex model structures (VIFs ≤ 5 recommended, e.g., James et al., 2023; Kim, 2019). Thus, we excluded the interaction terms with HRV, retaining only an HRV main effect among the other interaction effects with PA and BMI. The resulting model yielded no convergence failures and better values for multicollinearity in the Navon accuracy model (VIFs < 3.80) but also for the Navon RT model (VIFs < 3.03) and the Stroop RT (VIFs < 2.97) and accuracy (VIFs ≤ 5.10) models – which we deemed acceptable. Thus, we consistently fit this fixed effect structure across all (Navon vs. Stroop and RT vs. accuracy) DV's.

In equation form, the model was specified as:

$$\widehat{y_{ij}} = \beta_{0i} + \beta_{1i}\,congruency_{ij} + \beta_{2i}\,posture_{ij} + \beta_{3i}hrv_{ij} + \beta_{4i}PA_i + \beta_{5i}BMI_i$$
$$+ \beta_{6i}\,(congruency_{ij} \times posture_{ij}) + \beta_{7i}(congruency_{ij} \times PA_i)$$
$$+ \beta_{8i}(congruency_{ij} \times BMI_i) + \beta_{9i}\,(posture_{ij} \times PA_i)$$
$$+ \beta_{10i}(posture_{ij} \times BMI_i) + \beta_{11i}\,(congruency_{ij} \times posture_{ij} \times PA_i)$$
$$+ \beta_{12i}(congruency_{ij} \times posture_{ij} \times BMI_i)$$

where $\widehat{y_{ij}}$ is the predicted DV (Navon or Stroop RT or accuracy, respectively), with i indexing subjects and j indexing individual observations, β_{0i} is the intercept, hrv_{ij} is the (scaled) heart rate variability measure, $congruency_{ij}$ and $posture_{ij}$ are effect-coded categorical predictors (congruent vs. incongruent, sitting vs. standing), and PA_i and BMI_i are the (scaled) measures of physical activity and body mass index, respectively. If not mentioned otherwise (see selection of random effects below), the slope parameters were fixed effects only, i.e., for example $\beta_{4i} = \beta_4$. Because numerical values of VIFs may be affected by the choice of contrast coding (regardless of actual unchanged underlying collinearity), this model selection procedure was conducted based on treatment contrast coded categorical variables (to also allow appropriate fitting of respective control models as reported in Supplementary Material 7) and simultaneously ensured adequacy for models with sum contrast coded categorical variables as evident in subsequent checks (all VIFs < 5).

We then turned to the selection of appropriate random effect structures to account for possible cluster-induced correlations in the data. For each of the four models, we identified an appropriate model structure through a stepwise process based on likelihood ratio tests using the package *buildmer* (Voeten, 2025).

For the model using Stroop RT as the dependent variable (DV), this procedure suggested the inclusion of by-participant random intercepts and by-participant random slopes for the main effect of posture:

$$\beta_{0i} = \beta_0 + u_{0i}$$

$$\beta_{2i} = \beta_2 + u_{2i}$$

Where β_0 and β_2 are the fixed effect intercept and slope, respectively, and u_{0i} and u_{2i} are the by-participant random effects (equivalent for the upcoming equations below).

For the model using Stroop accuracy as the DV, this procedure suggested the inclusion of only by-participant random intercepts:

$$\beta_{0i} = \beta_0 + u_{0i}$$

For the model using Navon RT as the DV, this procedure suggested the inclusion of by-participant random intercepts, and by-participant random slopes for the main effects of posture, congruency, and BMI:

$$\beta_{0i} = \beta_0 + u_{0i}$$

$$\beta_{1i} = \beta_1 + u_{1i}$$

$$\beta_{2i} = \beta_2 + u_{2i}$$

$$\beta_{5i} = \beta_5 + u_{5i}$$

For the model using Navon accuracy as the DV, this procedure suggested the inclusion of by-participant random intercepts and by-participant random slopes for the main effect of congruency:

$$\beta_{0i} = \beta_0 + u_{0i}$$

$$\beta_{1i} = \beta_1 + u_{1i}$$

5. Exploratory Bayesian Analysis of Stroop and Navon RT

To test if we found evidence *against* an influence of posture on the Stroop RT effect (the classic effect reported in the original study by Rosenbaum et al., 2018), we exploratively ran an additional Bayesian LMM using the R package *brms* (Bürkner, 2017). To ensure consistency with previous work, we leaned strongly on the approach for the Bayesian analyses reported by Straub et al. (2022), using a shifted log-normal distribution for RT and the same priors (for details, we refer the reader to the original article of Straub et al., 2022; Appendix A). However, our model specifications deviated from those in Straub et al. (2022) as follows to ensure comparability with the frequentist statistics reported in our main text: We fit a model analyzing Stroop RT and including random intercepts and random slopes for the main effect of posture per subject and the three-way interactions of congruency x posture x PA, and congruency x posture x BMI, while controlling for the direct influence of HRV (see also Supplementary material 4 above)[1]. We estimated the posteriors using the NUTS sampler of STAN in *brms,* sampling eight independent Markov chains with 10000 iterations each from which every second sample was recorded (1000 warm-up, resulting in a total of 36000 post-warmup samples) to ensure precise posteriors. To improve sampling efficiency and address potential issues with divergent transitions, we set the maximum tree depth to 15 and the target acceptance probability (*adapt_delta*) to 0.99. These settings allowed for a more thorough exploration of the posterior distribution at the expense of longer computation times.

Rhat values ≤1.01 and visual inspection of chains ensured convergence. Models were validated with posterior predictive checks by sampling from the estimated parameters' posterior distributions before inspecting the overlayed observed data distribution with the simulated distributions. Posteriors were computed for each parameter using functions from

[1] The analysis scripts including the detailed model specifications will be made available on OSF upon acceptance of the manuscript. The OSF link will be included in the main manuscript.

the *bayestestR* package (Makowski et al., 2019). Table 5.1 shows the respective output for the Bayesian LMM on Stroop RT.

We then ran the equivalent model for Navon RT (where our frequentist analysis detected a significant modulation of the congruency effect by posture) using the same settings as above. Again, Rhat values ≤1.01 and visual inspection of chains ensured convergence, and models were validated with posterior predictive checks. Posteriors for this model are displayed in Table 5.2 for the Bayesian LMM on Navon RT. We found a highly credible interaction effect of posture and congruency, with 97% of the posterior mass pointing towards a negative effect estimate. To corroborate this finding, we ran prior sensitivity checks (Schad et al., 2023; see also Kreis et al., 2025 for a comparable approach) with varying standard deviations for the Gaussian prior around the null for the parameter estimates. Across several prior settings (SD's 0.5, 0.01, 0.001, 0.0001; in contrast to 0.2 used in the main analysis), the effect remained highly credible (see Table 5.3).

Table 5.1

Posteriors for Stroop Model Parameters

Parameter	*Median*	CI_{low}	CI_{high}	*pd*	*Rhat*	*ESS*
(Intercept)	0.3475	0.2589	0.4385	1.0000	1.0003	21894.8736
HRV	0.0038	-0.0247	0.0327	0.6041	1.0001	22647.0684
Congruency [incong.]	0.0338	0.0281	0.0396	1.0000	1.0002	30818.7556
Posture [stand]	-0.0011	-0.0189	0.0169	0.5481	1.0001	25203.0649
Physical Activity (PA)	-0.0117	-0.0580	0.0354	0.6961	1.0002	21732.8223
BMI	0.0178	-0.0300	0.0648	0.7731	1.0003	21142.4699
Congruency [incong.] × Posture [stand]	0.0009	-0.0043	0.0062	0.6378	0.9999	30421.6979
Congruency [incong.] × PA	-0.0032	-0.0085	0.0020	0.8841	1.0001	31544.0705
Posture [stand] × PA	0.0146	-0.0019	0.0315	0.9595	1.0000	26996.5534
Congruency [incong.] × BMI	0.0013	-0.0040	0.0065	0.6897	1.0001	32469.3264
Posture [stand] × BMI	0.0065	-0.0097	0.0227	0.7926	1.0000	26688.2379
(Congruency [incong.] × Posture [stand]) × PA	0.0030	-0.0022	0.0082	0.8670	1.0001	30426.1744
(Congruency [incong.] × Posture [stand]) × BMI	0.0017	-0.0034	0.0069	0.7482	1.0000	30441.3294

Note. CI = credibility interval; pd = probability of direction; ESS = effective sample size.

Table 5.2

Posteriors for Navon Model Parameters

Parameter	*Median*	CI_{low}	CI_{high}	*pd*	*Rhat*	*ESS*
(Intercept)	0.1319	0.0452	0.2167	0.9983	1.0001	22542.6538
HRV	0.0022	-0.0337	0.0410	0.5489	1.0002	19835.2525
Congruency [incong.]	0.0459	0.0316	0.0606	1.0000	1.0000	29539.1599
Posture [stand]	-0.0034	-0.0282	0.0210	0.6114	1.0000	21664.3738
Physical Activity (PA)	-0.0365	-0.0823	0.0143	0.9229	1.0001	18079.0274
BMI	0.0493	-0.0442	0.1503	0.8676	1.0002	24718.9558
Congruency [incong.] × Posture [stand]	-0.0049	-0.0101	0.0002	0.9708	1.0002	32573.7711
Congruency [incong.] × PA	-0.0016	-0.0158	0.0126	0.5873	1.0000	32843.5031
Posture [stand] × PA	0.0090	-0.0141	0.0324	0.7846	1.0002	25302.9024
Congruency [incong.] × BMI	0.0002	-0.0136	0.0141	0.5122	1.0001	31479.6293
Posture [stand] × BMI	-0.0000	-0.0219	0.0221	0.5001	1.0000	26679.7997
(Congruency [incong.] × Posture [stand]) × PA	0.0034	-0.0016	0.0085	0.9037	1.0000	32548.5347
(Congruency [incong.] × Posture [stand]) × BMI	-0.0018	-0.0068	0.0031	0.7657	1.0002	32445.0018

Note. CI = credibility interval; pd = probability of direction; ESS = effective sample size.

Table 5.3

Prior sensitivity checks using varying standard deviations for the Gaussian priors around the null in the Navon RT model

Parameter	*Prior SD*	*Posterior Median*	*pd*	*Rhat*	*ESS*
Congruency [incong.] × Posture [stand]	0.5	-0.0049	0.9700	1.0001	31011.2824
Congruency [incong.] × Posture [stand]	0.2	-0.0049	0.9697	1.0000	31075.1040
Congruency [incong.] × Posture [stand]	0.01	-0.0038	0.9797	1.0000	33738.1358
Congruency [incong.] × Posture [stand]	0.001	-0.0009	0.9884	1.0001	35663.2004
Congruency [incong.] × Posture [stand]	0.0001	-0.0002	0.9875	1.0001	35577.8534

Note. pd = probability of direction; ESS = effective sample size.

6. Control analyses with continuous covariates centered within-cluster

An alternative way to center continuous variables, opposed to centering on the grand mean (CGM), as we did in our main analysis, is to center the variable within each cluster (CWC, e.g., within each posture condition). Some people argue that the choice of centering approach can affect both the value and interpretation of parameters, as well as their statistical significance (e.g., Enders & Tofighi, 2007). Here, we report an alternative control analysis that uses CWC for all continuous covariates.

Table 6.1

Posture Modulation of Stroop Congruency Effects

	**Stroop log-RT**		**Stroop Acc.**	
Predictors	*Estimates*	*p*	*Odds Ratios*	*p*
(Intercept)	6.40 *** (6.33 – 6.47)	**<0.001**	41.30 (29.92 – 57.00)	**<0.001**
HRV	0.00 (-0.04 – 0.04)	0.880	1.01 (0.78 – 1.31)	0.957
Congruency [incong.]	0.05 *** (0.04 – 0.06)	**<0.001**	0.78 (0.67 – 0.91)	**0.002**
Posture [stand]	-0.00 (-0.03 – 0.02)	0.850	1.06 (0.91 – 1.24)	0.448
Physical Activity (PA)	-0.01 (-0.08 – 0.06)	0.783	0.96 (0.71 – 1.29)	0.774
BMI	0.02 (-0.05 – 0.09)	0.518	0.85 (0.63 – 1.16)	0.313
Congruency [incong.] × Posture [stand]	0.00 (-0.01 – 0.01)	0.602	0.91 (0.78 – 1.06)	0.231
Congruency [incong.] × PA	-0.00 (-0.01 – 0.00)	0.358	0.96 (0.83 – 1.11)	0.619
Posture [stand] × PA	0.02 (-0.00 – 0.04)	0.109	1.07 (0.93 – 1.24)	0.353
Congruency [incong.] × BMI	0.00 (-0.00 – 0.01)	0.485	0.98 (0.85 – 1.12)	0.736
Posture [stand] × BMI	0.01 (-0.01 – 0.03)	0.449	0.91 (0.79 – 1.05)	0.184
(Congruency [incong.] × Posture [stand]) × PA	0.00 (-0.00 – 0.01)	0.244	0.83 (0.72 – 0.96)	**0.015**
(Congruency [incong.] × Posture [stand]) × BMI	0.00 (-0.01 – 0.01)	0.501	0.93 (0.81 – 1.07)	0.307
**Random Effects**				
σ^2	0.09		3.29	
τ_{00}	0.04 $_{sb}$		0.60 $_{sb}$	
τ_{11}	0.00 $_{sb.posture1}$			

ρ_{01}	0.09 sb	
ICC	0.35	0.15
N	36 sb	36 sb
Observations	5573	5760
Marginal R^2 / Conditional R^2	0.027 / 0.363	0.039 / 0.187

Note. Categorical predictors *congruency* (congruent = -1; incongruent = 1) and *posture* (sit = -1; stand = 1) were effect-coded (by applying sum contrasts). Consequently, all effects can be interpreted as main effects relative to the grand average across all conditions and covariate levels (for example, coefficients for the predictor "Congruency [incong.]" describe the main effect of incongruent trial conditions, coefficients for the predictor "Posture [stand]" describe the main effect for the standing posture condition). All continuous predictor variables were z-standardized before inclusion into the models. Estimates for RT-models thus represent the change in log-RT when the predictor variable increases by 1 SD while holding other variables constant; Estimates of the Accuracy-models represent the change in the Odds Ratio for responding correctly when the predictor variable increases by 1 SD while holding other variables constant. Values in parentheses refer to the 95% confidence interval. Satterthwaite's method is used to approximate the degrees of freedom for the LMMs, and Wald's method is used for the GLMMs.

Table 6.2

Posture Modulation of Navon Congruency Effects

	Navon log-RT		Navon Acc.	
Predictors	*Estimates*	*p*	*Odds Ratios*	*p*
(Intercept)	6.45<br>(6.40 – 6.50)	**<0.001**	18.58<br>(12.02 – 28.72)	**<0.001**
HRV	-0.01<br>(-0.04 – 0.02)	0.614	1.12<br>(0.87 – 1.43)	0.377
Congruency [incong.]	0.05<br>(0.03 – 0.06)	**<0.001**	0.66<br>(0.52 – 0.85)	**0.001**
Posture [stand]	-0.00<br>(-0.03 – 0.02)	0.784	1.13<br>(1.01 – 1.26)	**0.031**
Physical Activity (PA)	-0.04<br>(-0.08 – 0.00)	0.058	1.05<br>(0.68 – 1.62)	0.813
BMI	0.06<br>(-0.07 – 0.19)	0.317	0.96<br>(0.62 – 1.49)	0.849
Congruency [incong.] × Posture [stand]	-0.01<br>(-0.01 – -0.00)	**0.019**	0.96<br>(0.86 – 1.07)	0.426
Congruency [incong.] × PA	-0.00<br>(-0.02 – 0.01)	0.650	1.26<br>(1.00 – 1.58)	**0.046**
Posture [stand] × PA	0.01<br>(-0.01 – 0.04)	0.234	0.95<br>(0.85 – 1.06)	0.359
Congruency [incong.] × BMI	0.00<br>(-0.01 – 0.02)	0.758	1.03<br>(0.82 – 1.30)	0.779
Posture [stand] × BMI	-0.00<br>(-0.03 – 0.02)	0.895	1.02<br>(0.91 – 1.15)	0.700
(Congruency [incong.] × Posture [stand]) × PA	0.00<br>(-0.00 – 0.01)	0.202	0.99<br>(0.89 – 1.11)	0.909
(Congruency [incong.] × Posture [stand]) × BMI	-0.00<br>(-0.01 – 0.00)	0.424	0.96<br>(0.85 – 1.08)	0.460
**Random Effects**				
σ^2	0.04		3.29	
τ_{00}	0.01 $_{sb}$		1.47 $_{sb}$	
τ_{11}	0.00 $_{sb.posture1}$			

	0.00 sb.congruent1	0.27 sb.congruent1
	0.08 sb.bmi_scaled	
ρ_{01}	-0.03	-0.18 sb
	0.37	
	0.66	
ICC	0.32	0.35
N	36 sb	36 sb
Observations	3972	4395
Marginal R^2 / Conditional R^2	0.136 / 0.412	0.050 / 0.379

Note. Categorical predictors *congruency* (congruent = -1; incongruent = 1) and *posture* (sit = -1; stand = 1) were effect-coded (by applying sum contrasts). Consequently, all effects can be interpreted as main effects relative to the grand average across all conditions and covariate levels (for example, coefficients for the predictor “Congruency [incong.]“ describe the main effect of incongruent trial conditions, coefficients for the predictor “Posture [stand]“ describe the main effect for the standing posture condition). All continuous predictor variables were z-standardized before inclusion into the models. Estimates for RT-models thus represent the change in log-RT when the predictor variable increases by 1 SD while holding other variables constant; Estimates of the Accuracy-models represent the change in the Odds Ratio for responding correctly when the predictor variable increases by 1 SD while holding other variables constant. Values in parentheses refer to the 95% confidence interval. Satterthwaite’s method is used to approximate the degrees of freedom for the LMMs, and Wald’s method is used for the GLMMs.

7. Control analyses with treatment contrasts

The choice of contrasts (treatment contrast vs. sum contrast) for categorical predictors plays a central role in interpreting coefficients and determining which effects appear to be significant (Schad et al., 2020; Brehm & Alday, 2022; Singmann & Kellen, 2019). Here, we report an alternative control analysis that uses treatment contrasts instead of sum contrasts.

Table 7.1

Posture Modulation of Stroop Congruency Effects

	Stroop log-RT		Stroop Acc.	
Predictors	*Estimates*	*p*	*Odds Ratios*	*p*
(Intercept)	6.35<br>(6.28 – 6.43)	**<0.001**	45.07<br>(29.40 – 69.09)	<0.001
HRV	0.00<br>(-0.04 – 0.04)	0.892	1.01<br>(0.77 – 1.32)	0.955
Congruency [incong.]	0.10<br>(0.08 – 0.12)	**<0.001**	0.74<br>(0.49 – 1.13)	0.162
Posture [stand]	-0.01<br>(-0.06 – 0.05)	0.781	1.37<br>(0.84 – 2.23)	0.213
Physical Activity (PA)	-0.02<br>(-0.09 – 0.05)	0.586	0.77<br>(0.53 – 1.12)	0.167
BMI	0.01<br>(-0.06 – 0.09)	0.705	0.89<br>(0.59 – 1.35)	0.595
Congruency [incong.] × Posture [stand]	0.01<br>(-0.02 – 0.04)	0.602	0.69<br>(0.37 – 1.27)	0.231
Congruency [incong.] × PA	-0.02<br>(-0.04 – 0.01)	0.143	1.34<br>(0.92 – 1.95)	0.125
Posture [stand] × PA	0.03<br>(-0.02 – 0.08)	0.260	1.66<br>(1.04 – 2.65)	**0.035**
Congruency [incong.] × BMI	0.00<br>(-0.02 – 0.02)	0.986	1.10<br>(0.73 – 1.66)	0.640
Posture [stand] × BMI	0.01<br>(-0.04 – 0.06)	0.623	0.96<br>(0.62 – 1.48)	0.841
(Congruency [incong.] × Posture [stand]) × PA	0.02<br>(-0.01 – 0.05)	0.244	0.48<br>(0.27 – 0.86)	**0.014**
(Congruency [incong.] × Posture [stand]) × BMI	0.01<br>(-0.02 – 0.04)	0.501	0.75<br>(0.43 – 1.31)	0.307
**Random Effects**				
σ^2	0.09		3.29	
τ_{00}	0.04 $_{sb}$		0.60 $_{sb}$	
τ_{11}	0.02 $_{sb.postureStanding}$			

ρ_{01}	-0.22 $_{sb}$	
ICC	0.35	0.15
N	36 $_{sb}$	36 $_{sb}$
Observations	5573	5760
Marginal R^2 / Conditional R^2	0.027 / 0.363	0.039 / 0.187

Note. All continuous predictor variables were z-standardized before inclusion into the models. Estimates for RT-models thus represent the change in log-RT when the predictor variable increases by 1 SD while holding other variables constant; Estimates of the Accuracy-models represent the change in the Odds Ratio for responding correctly when the predictor variable increases by 1 SD while holding other variables constant. Values in parentheses refer to the 95% confidence interval. Satterthwaite's method is used to approximate the degrees of freedom for the LMM's, Wald's method is used for the GLMM's.

Table 7.2

Posture Modulation of Navon Congruency Effects

	Navon log-RT		Navon Acc.	
Predictors	*Estimates*	*p*	*Odds Ratios*	*p*
(Intercept)	6.40<br>(6.34 – 6.46)	**<0.001**	23.09<br>(13.04 – 40.88)	**<0.001**
HRV	-0.01<br>(-0.04 – 0.02)	0.609	1.11<br>(0.87 – 1.43)	0.404
Congruency [incong.]	0.11<br>(0.08 – 0.14)	**<0.001**	0.48<br>(0.28 – 0.82)	**0.007**
Posture [stand]	0.00<br>(-0.05 – 0.06)	0.881	1.47<br>(1.02 – 2.11)	**0.039**
Physical Activity (PA)	-0.05<br>(-0.10 – 0.00)	0.073	0.88<br>(0.51 – 1.50)	0.633
BMI	0.06<br>(-0.07 – 0.19)	0.346	0.86<br>(0.50 – 1.51)	0.606
Congruency [incong.] × Posture [stand]	-0.03<br>(-0.05 – -0.00)	**0.019**	0.84<br>(0.54 – 1.31)	0.432
Congruency [incong.] × PA	-0.01<br>(-0.05 – 0.02)	0.361	1.60<br>(0.97 – 2.65)	0.065
Posture [stand] × PA	0.02<br>(-0.03 – 0.07)	0.401	0.91<br>(0.65 – 1.28)	0.601
Congruency [incong.] × BMI	0.01<br>(-0.02 – 0.04)	0.551	1.17<br>(0.71 – 1.93)	0.539
Posture [stand] × BMI	0.00<br>(-0.05 – 0.05)	0.943	1.15<br>(0.79 – 1.66)	0.467
(Congruency [incong.] × Posture [stand]) × PA	0.02<br>(-0.01 – 0.04)	0.202	0.97<br>(0.62 – 1.52)	0.908
(Congruency [incong.] × Posture [stand]) × BMI	-0.01<br>(-0.03 – 0.01)	0.423	0.83<br>(0.52 – 1.34)	0.456
**Random Effects**				
σ^2	0.04		3.29	
τ_{00}	0.01_{sb}		1.97_{sb}	
τ_{11}	$0.02_{\text{sb.postureStanding}}$			

	0.01 sb.congruentIncongruent	1.09 sb.congruentIncongruent
	0.08 sb.bmi_scaled	
ρ_{01}	-0.55	-0.53 sb
	0.11	
	0.27	
ICC	0.73	0.35
N	36 sb	36 sb
Observations	3972	4395
Marginal R^2 / Conditional R^2	0.059 / 0.746	0.049 / 0.379

Note. All continuous predictor variables were z-standardized before inclusion into the models. Estimates for RT-models thus represent the change in log-RT when the predictor variable increases by 1 SD while holding other variables constant; Estimates of the Accuracy-models represent the change in the Odds Ratio for responding correctly when the predictor variable increases by 1 SD while holding other variables constant. Values in parentheses refer to the 95% confidence interval. Satterthwaite's method is used to approximate the degrees of freedom for the LMM's, Wald's method is used for the GLMM's.
